# Supplementary material for: Role of Rac1 in p53-Related Proliferation and Drug Sensitivity in Multiple Myeloma
Source: Cancers (Basel). 2025 Jan 29;17(3):461. doi: 10.3390/cancers17030461 (PMC11815915; doi:10.3390/cancers17030461)

# Fig.1

## A KMS11 / Tet-on p53

p53

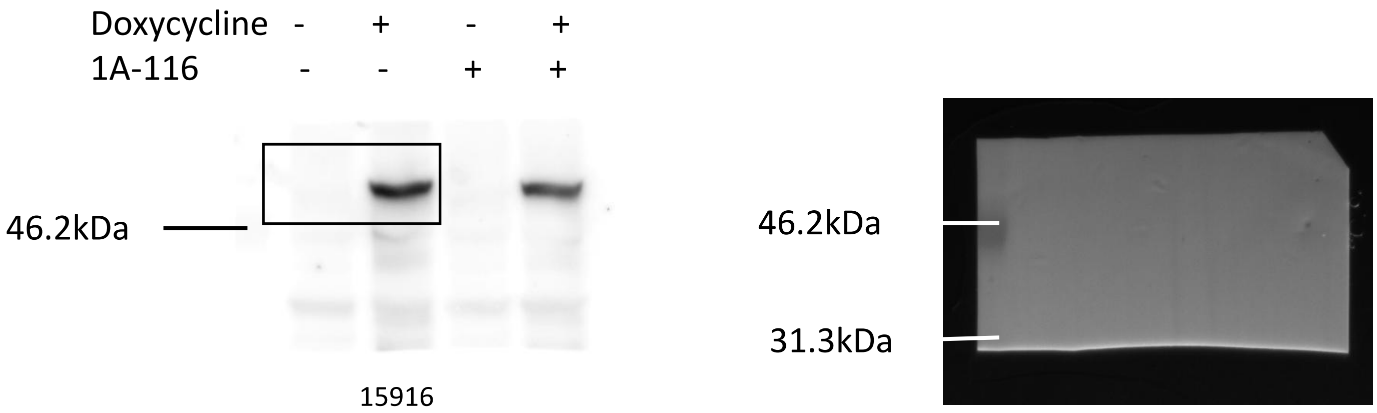


p21

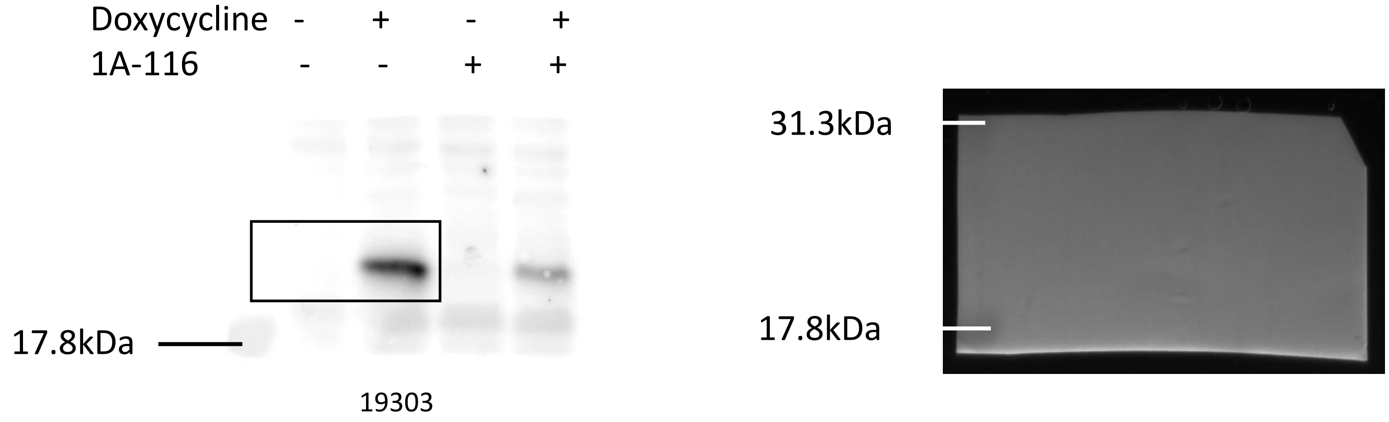


Western Blotting results in Figure1 A and B are part of results in Figure 3B.

Fig.1

# A KMS11 / Tet-on p53

Mdm2

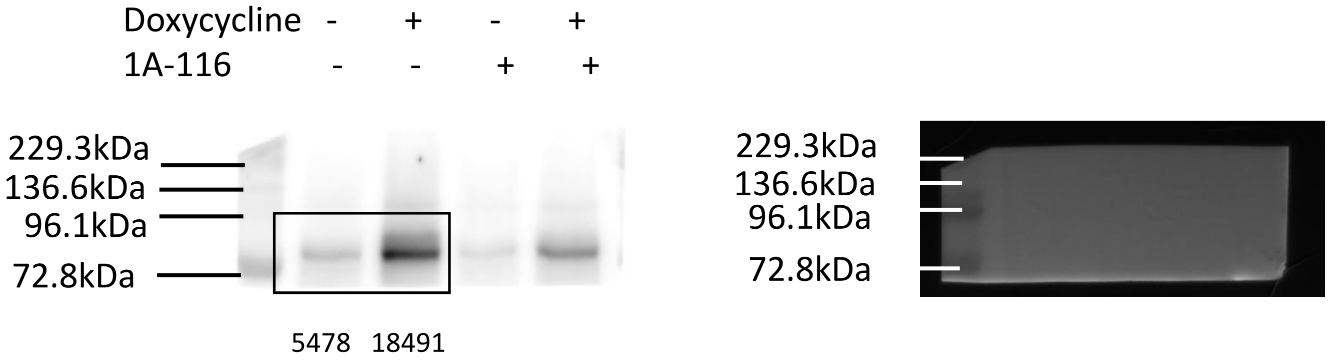


ACTB

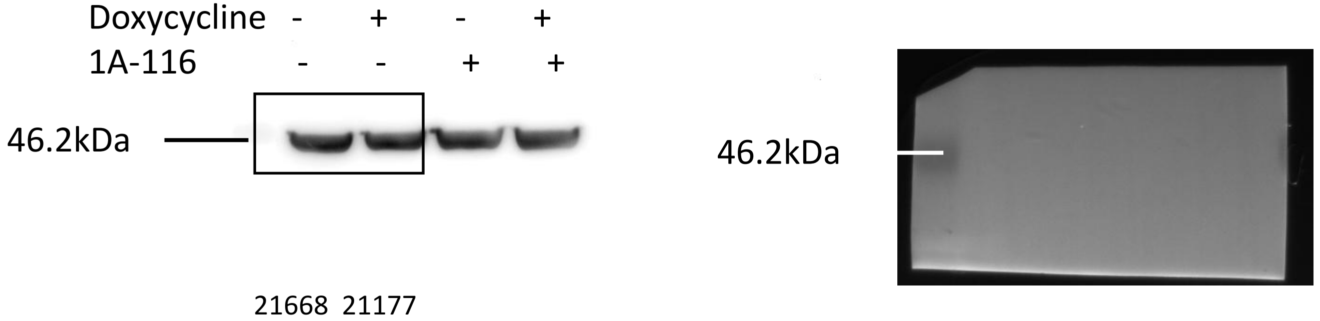


Western Blotting results in Figure1 A and B are part of results in Figure 3B.

# Fig.1

## B KMS26 / Tet-on p53

### p53

Doxycycline - + - +
1A-116 - - + +

46.2kDa

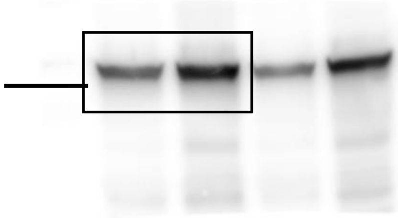

14330 21990

46.2kDa
31.3kDa

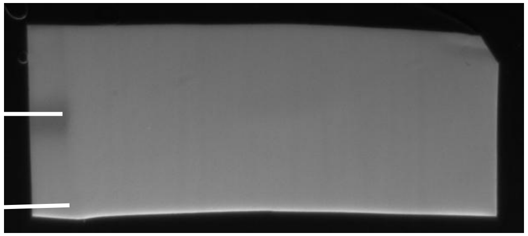

### p21

Doxycycline - + - +
1A-116 - - + +

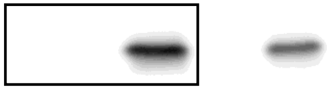

24535

31.3kDa
17.8kDa

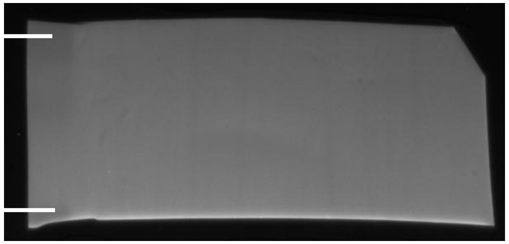

Western Blotting results in Figure1 A and B are part of results in Figure 3B.

Fig.1

# B KMS26 / Tet-on p53

## Mdm2

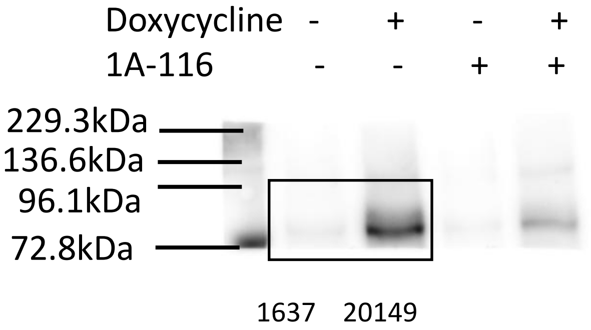


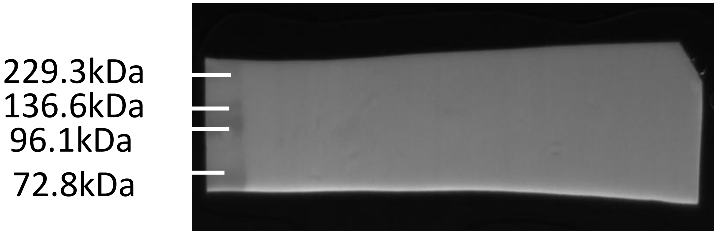


## ACTB

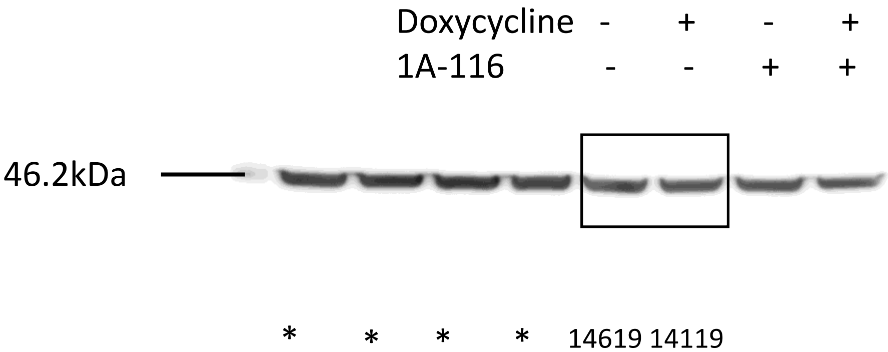


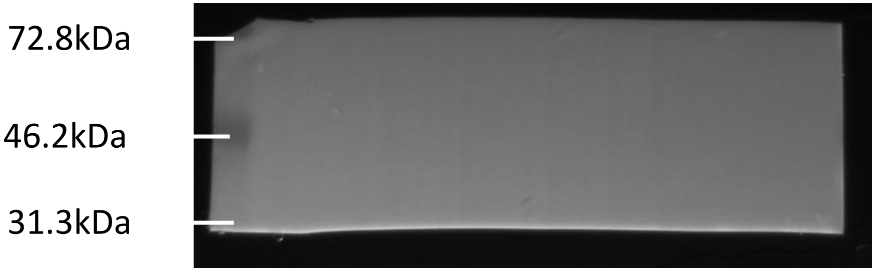


*; No used in results.

Western Blotting results in Figure1 A and B are part of results in Figure 3B.

# Fig.2F

Rac1

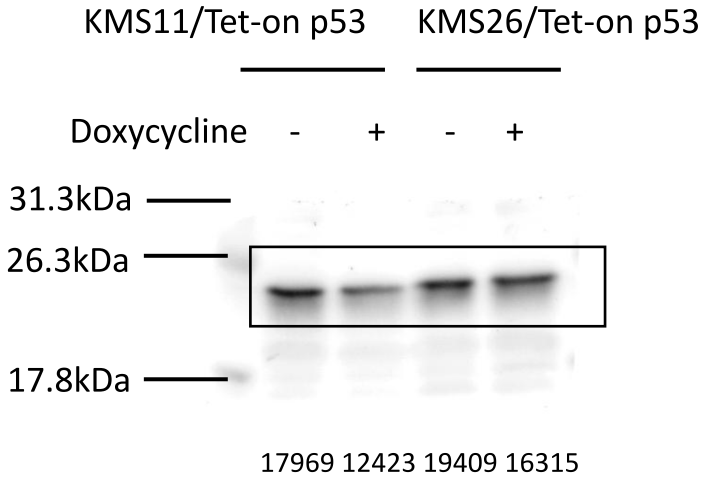
KMS11/Tet-on p53
KMS26/Tet-on p53
Doxycycline - + - +
31.3kDa
26.3kDa
17.8kDa
17969 12423 19409 16315

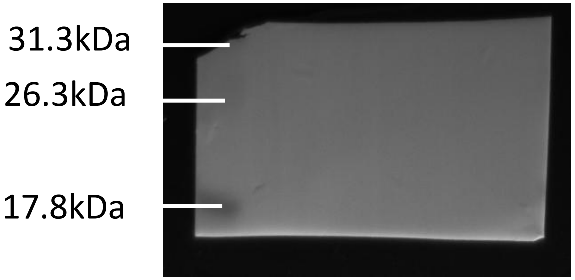
31.3kDa
26.3kDa
17.8kDa

# Fig.2F

p53

A

KMS11/Tet-on p53 KMS26/Tet-on p53

Doxycycline - + - +

72.8kDa

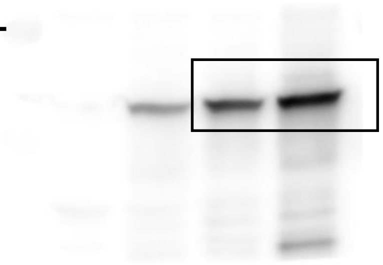

* * 11436 15117

B

KMS11/Tet-on p53 KMS26/Tet-on p53

Doxycycline - + - +

72.8kDa

46.2kDa

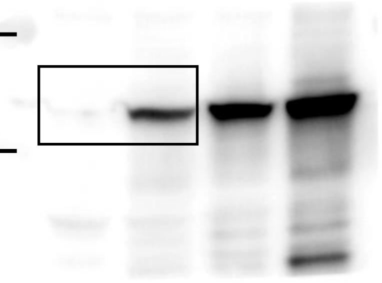

ND 12386 * *

72.8kDa

46.2kDa

31.3kDa

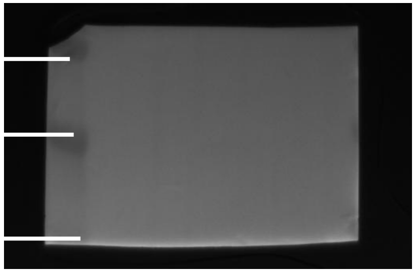

Detected on the same membrane with different exposure times.
Exposure time A: 40sec, B: 90sec.
ND; Not detected, *; No used in results.

Fig.2F

ACTB

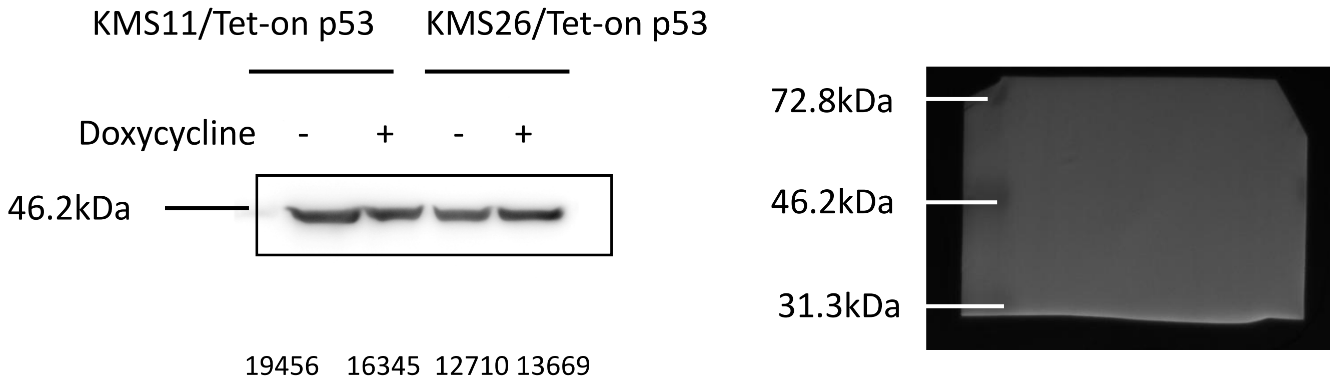
KMS11/Tet-on p53
KMS26/Tet-on p53
Doxycycline
-
+
-
+
46.2kDa
19456
16345
12710
13669
72.8kDa
46.2kDa
31.3kDa

Fig.2F MM.1S

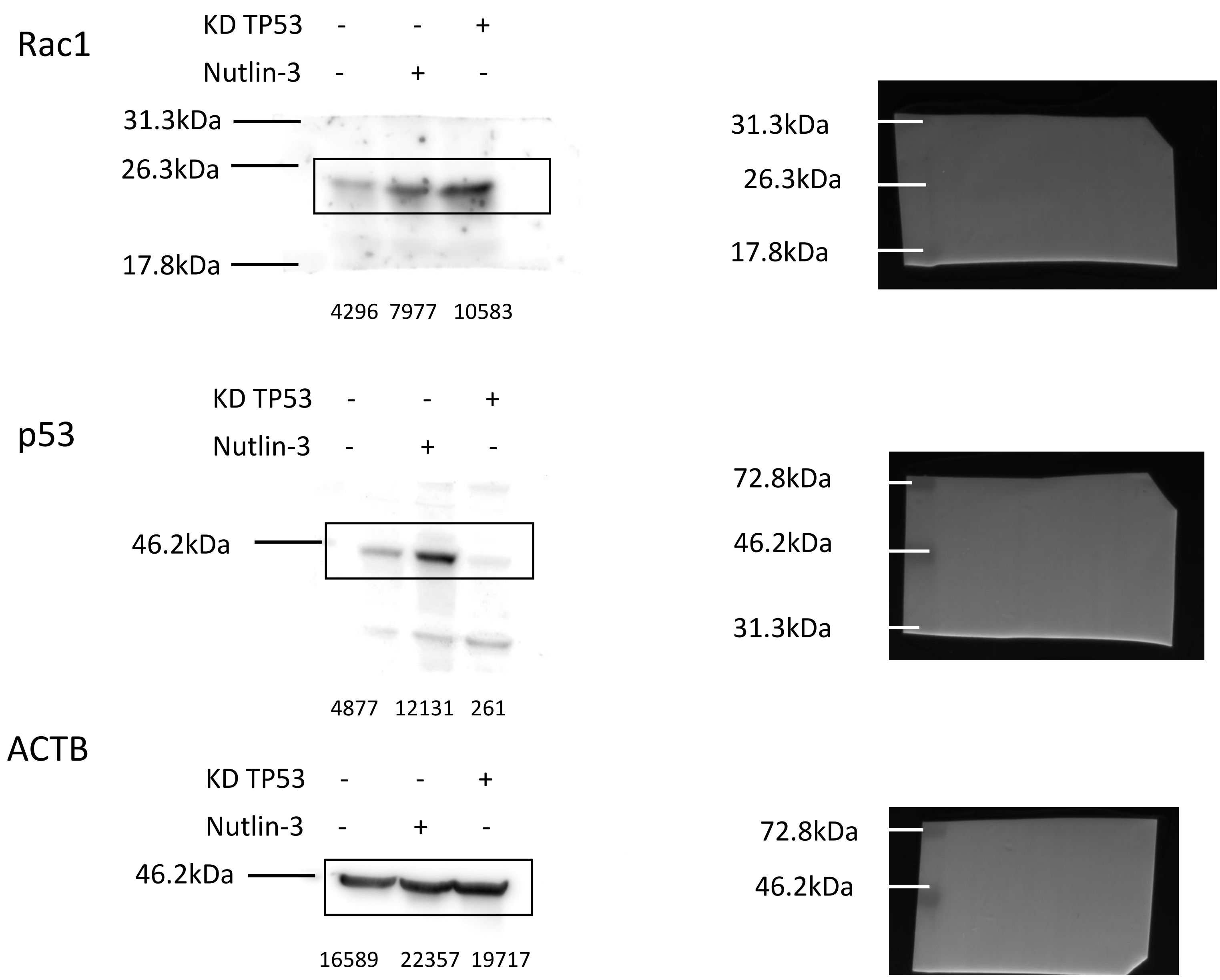
Rac1
KD TP53 - - +
Nutlin-3 - + -
31.3kDa
26.3kDa
17.8kDa
4296 7977 10583
31.3kDa
26.3kDa
17.8kDa
p53
KD TP53 - - +
Nutlin-3 - + -
46.2kDa
4877 12131 261
72.8kDa
46.2kDa
31.3kDa
ACTB
KD TP53 - - +
Nutlin-3 - + -
46.2kDa
16589 22357 19717
72.8kDa
46.2kDa

# Fig.3B

# KMS11 / Tet-on p53

## p53

| | | | | |
|---|---|---|---|---|
| Doxycycline | - | + | - | + |
| 1A-116 | - | - | + | + |

46.2kDa

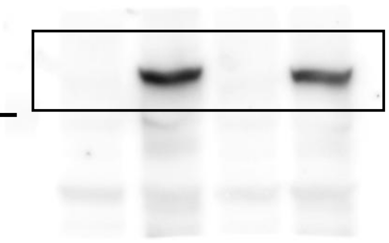

15916 11960

46.2kDa

31.3kDa

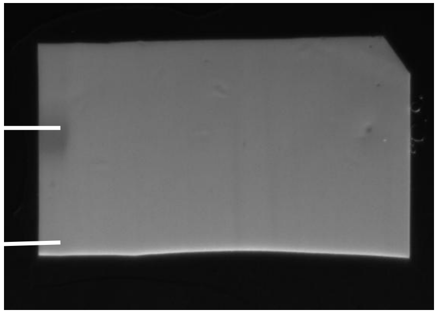

## p21

| | | | | |
|---|---|---|---|---|
| Doxycycline | - | + | - | + |
| 1A-116 | - | - | + | + |

17.8kDa

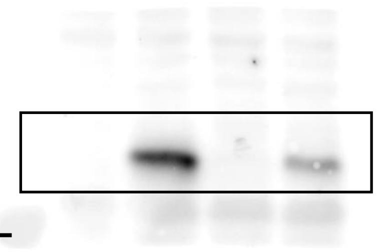

19303 5204

31.3kDa

17.8kDa

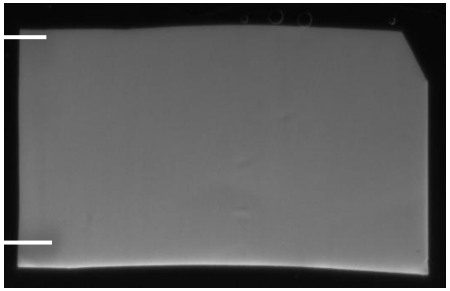

Fig.3B

KMS11 / Tet-on p53

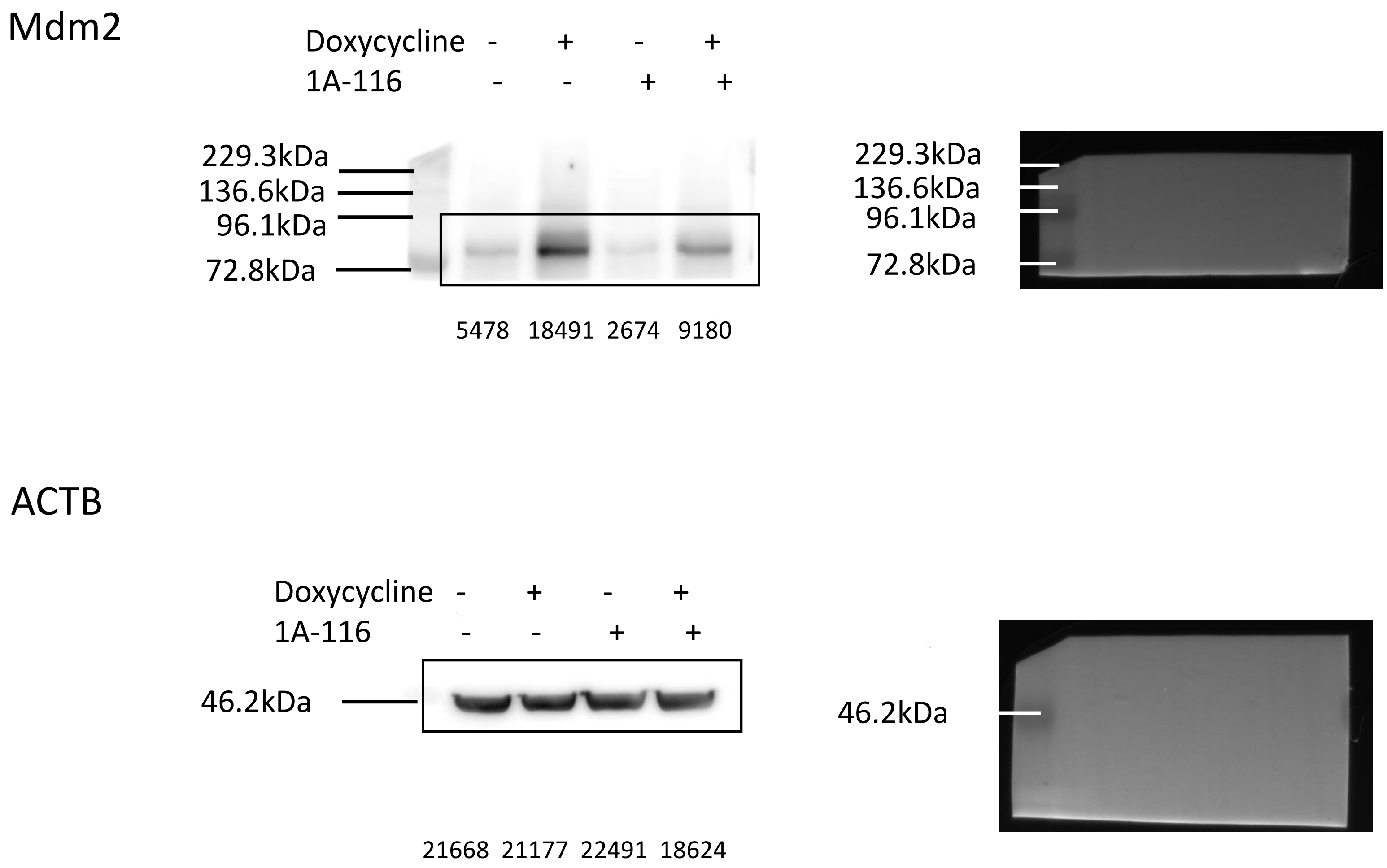

Fig.3B

# KMS26 / Tet-on p53

p53

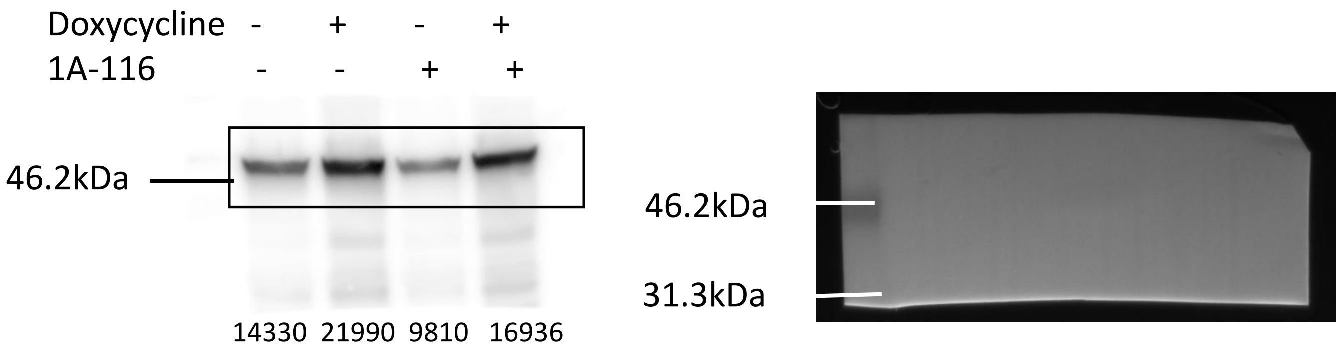


p21

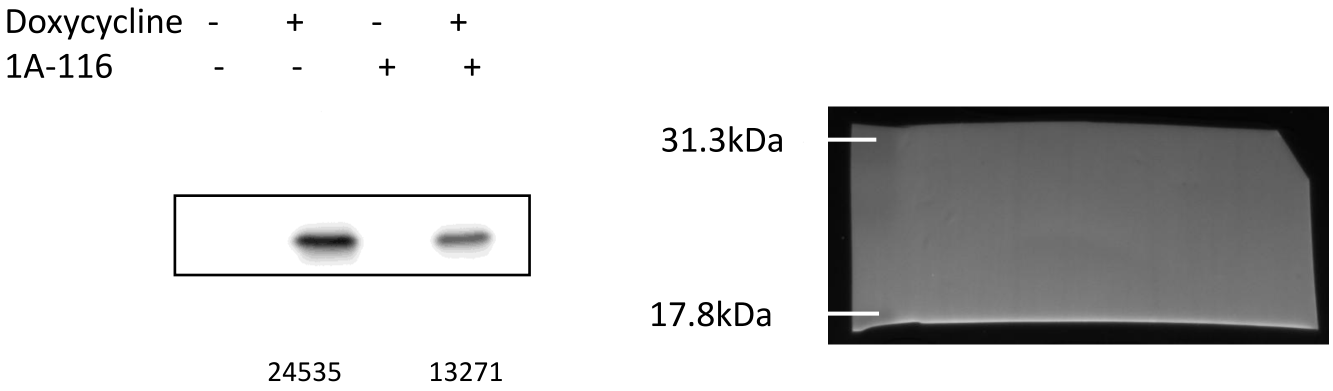

Fig.3B

# KMS26 / Tet-on p53

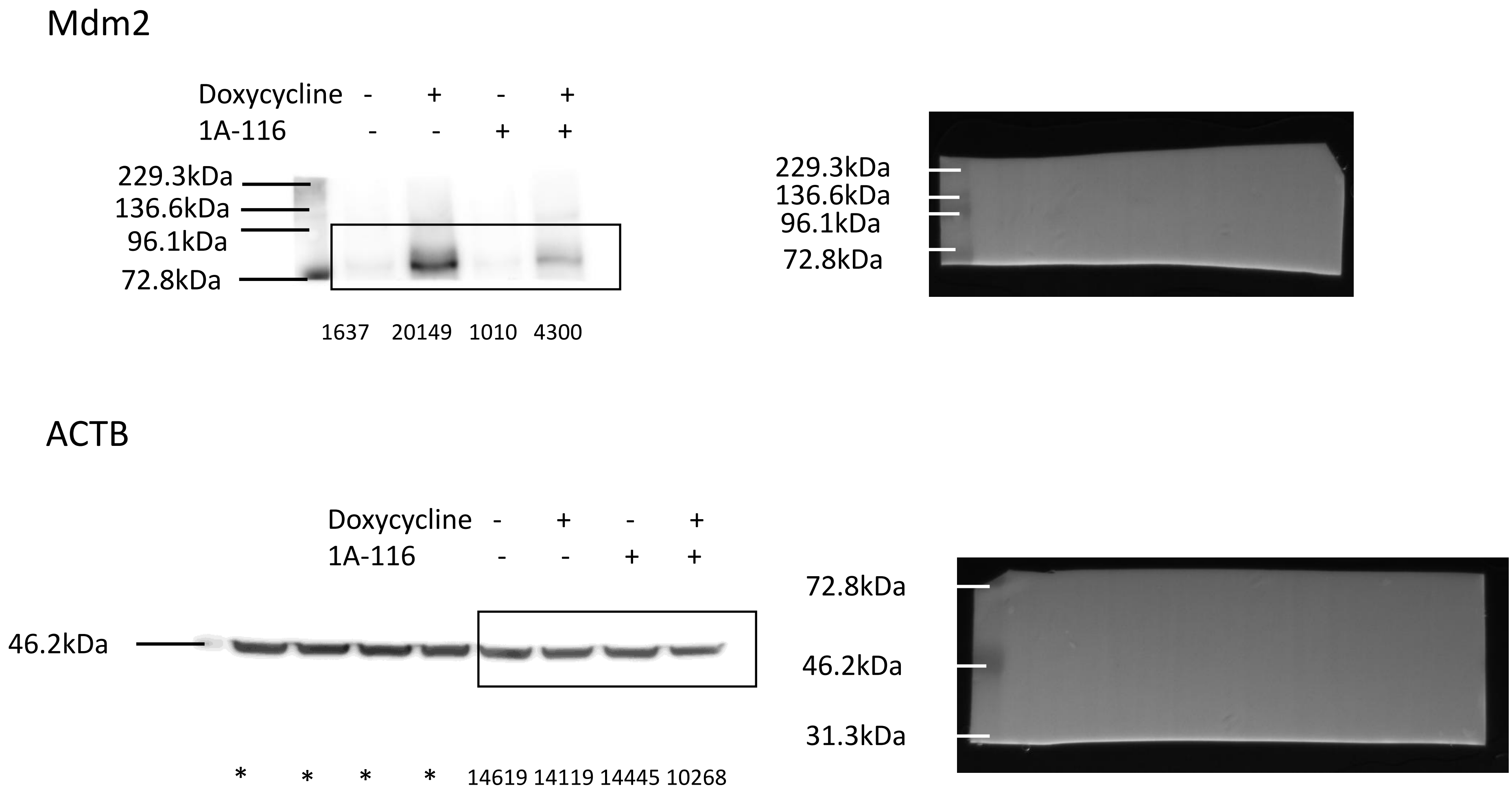


*; No used in results.

Fig.3B

MM.1S with Nutlin-3

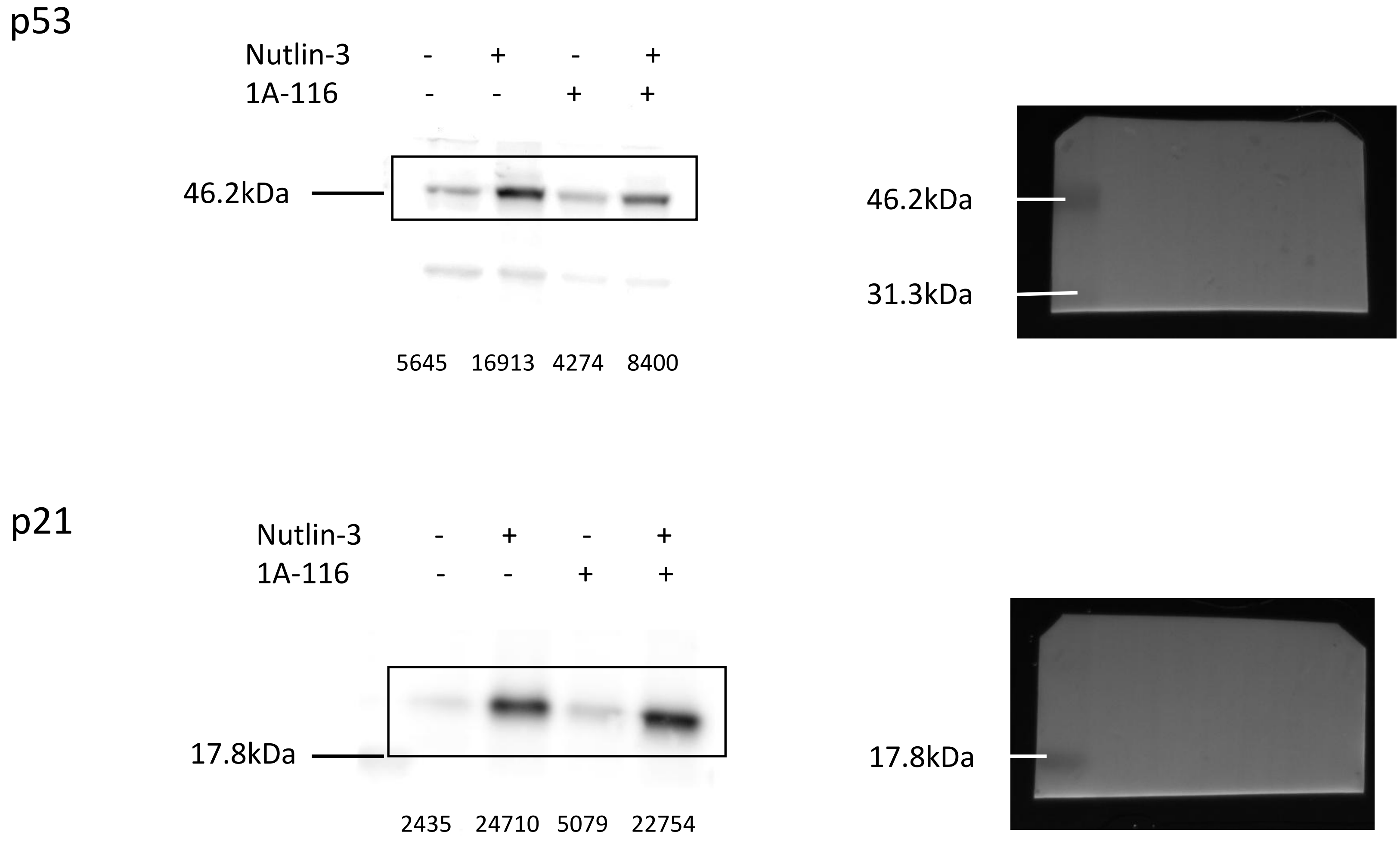

Fig.3B

# MM.1S with Nutlin-3

## Mdm2

| | | | | |
|---|---|---|---|---|
| Nutlin-3 | - | + | - | + |
| 1A-116 | - | - | + | + |

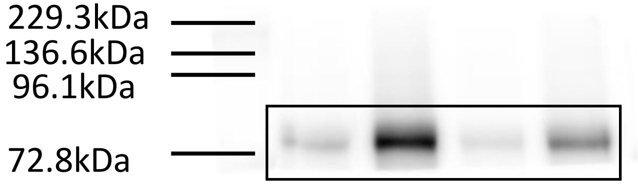


4739 18845 2033 8317

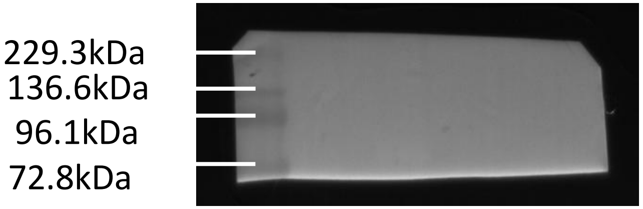


## ACTB

| | | | | |
|---|---|---|---|---|
| Nutlin-3 | - | + | - | + |
| 1A-116 | - | - | + | + |

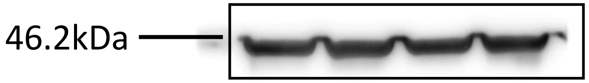


23614 25842 23736 24198

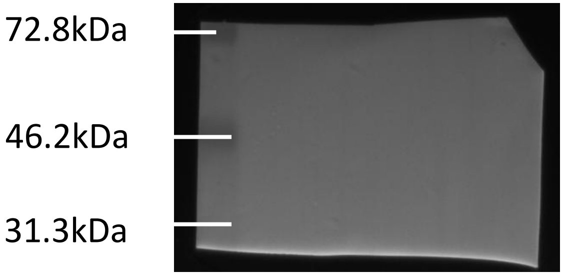

Fig.3B

# MM.1S with *TP53* knockdown

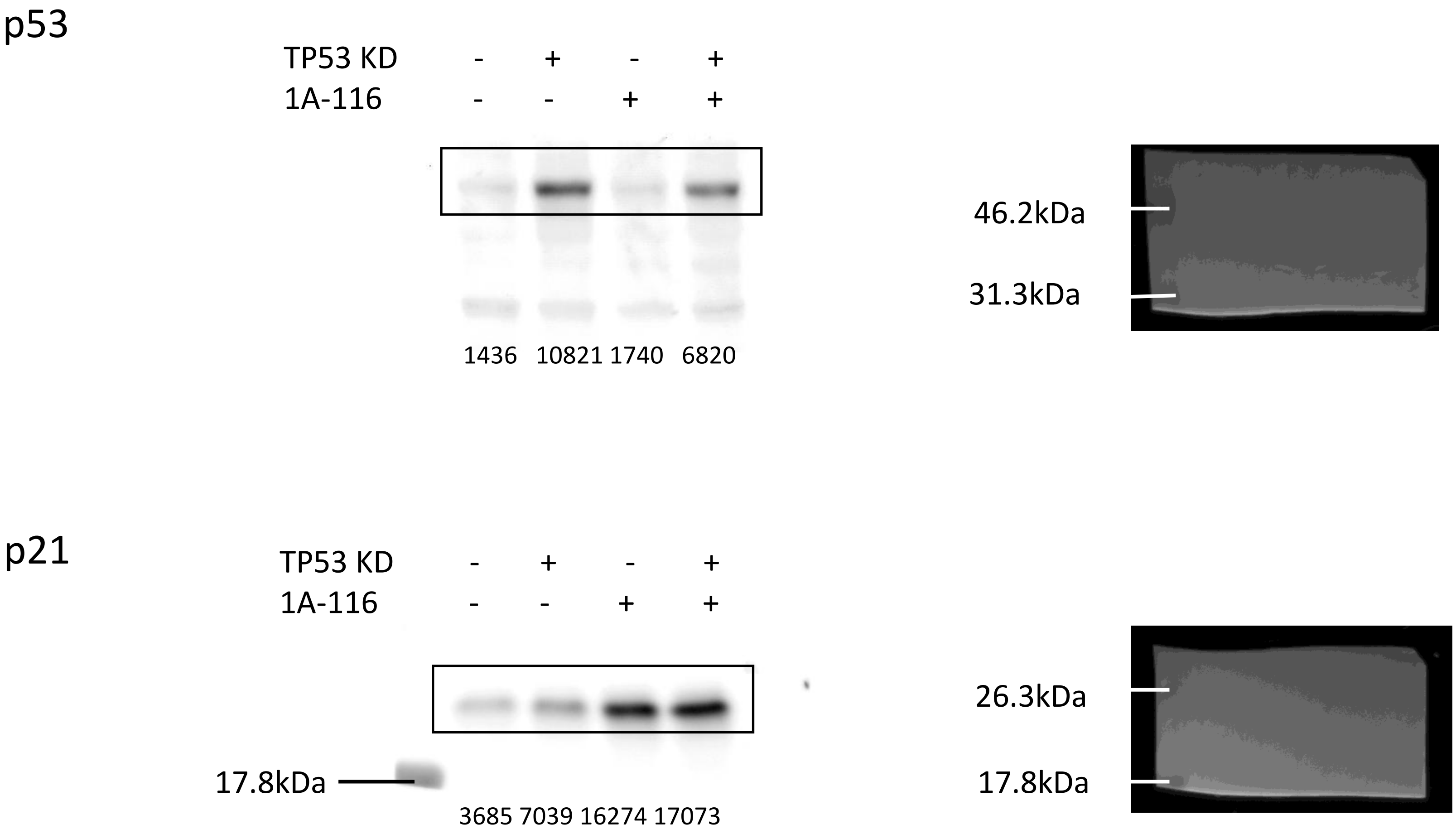


KD; knock down.

Fig.3B

# MM.1S with *TP53* knockdown

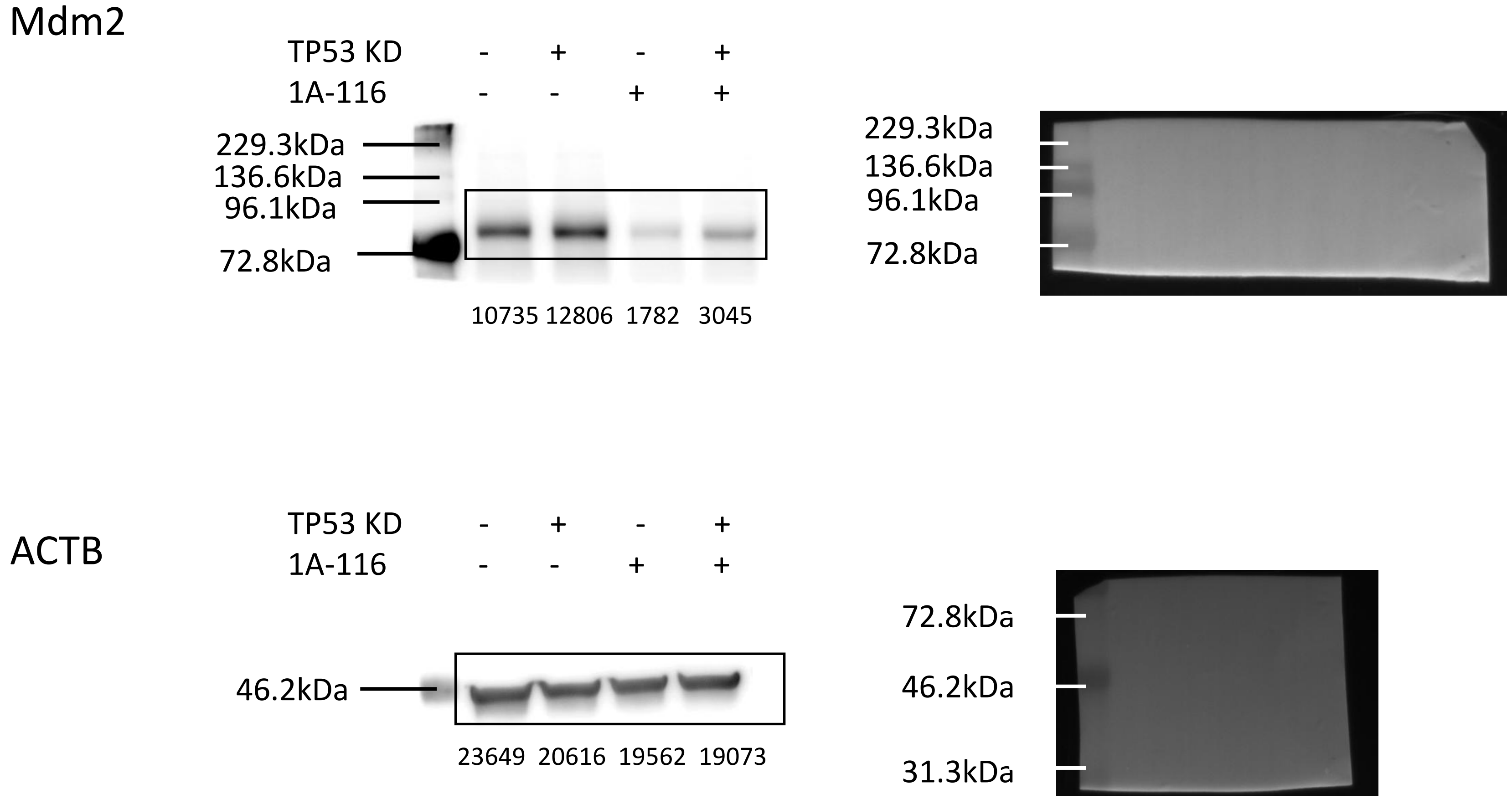

# Fig.S1 B

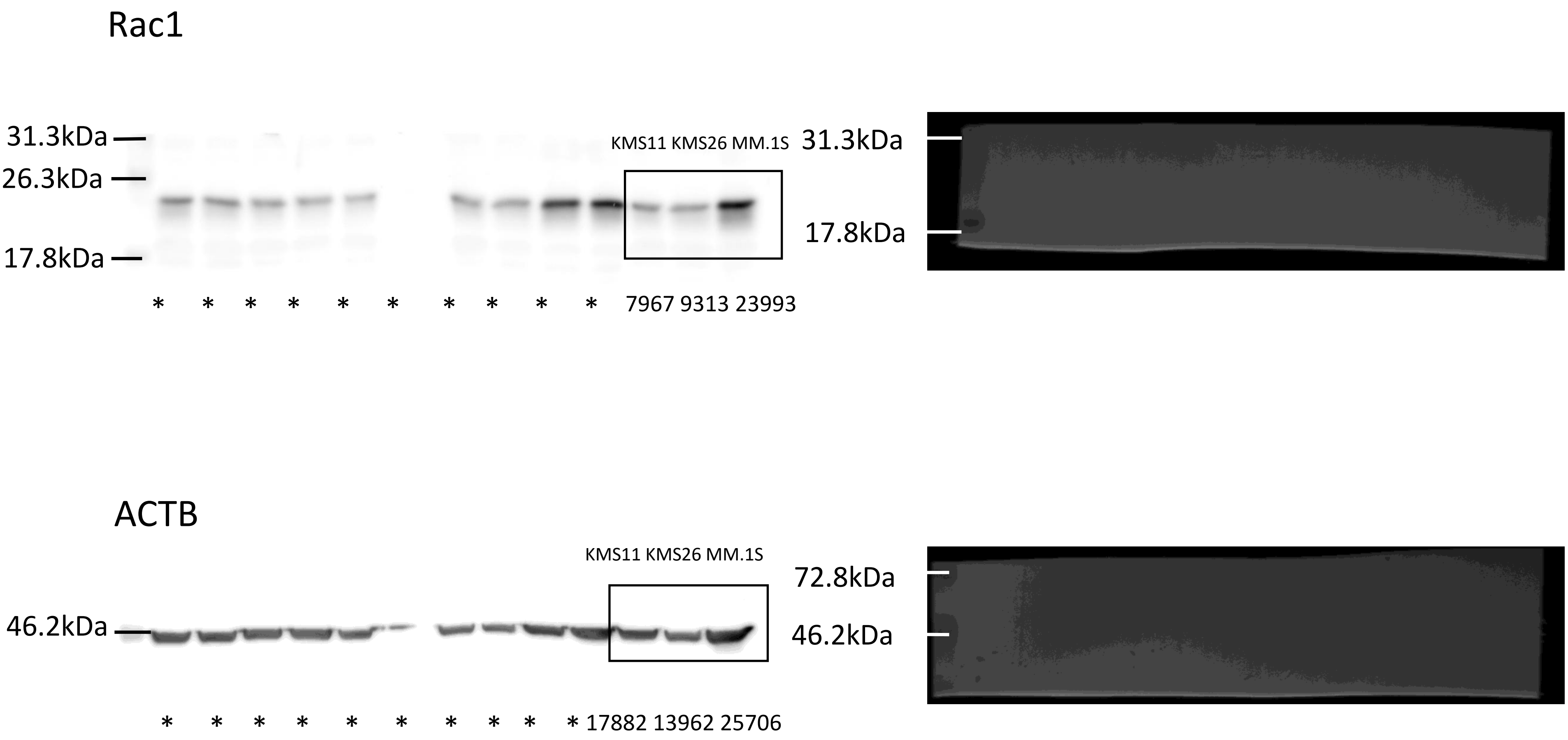


*; No used in results.

# Fig.S2 A,B 48h

## Rac1

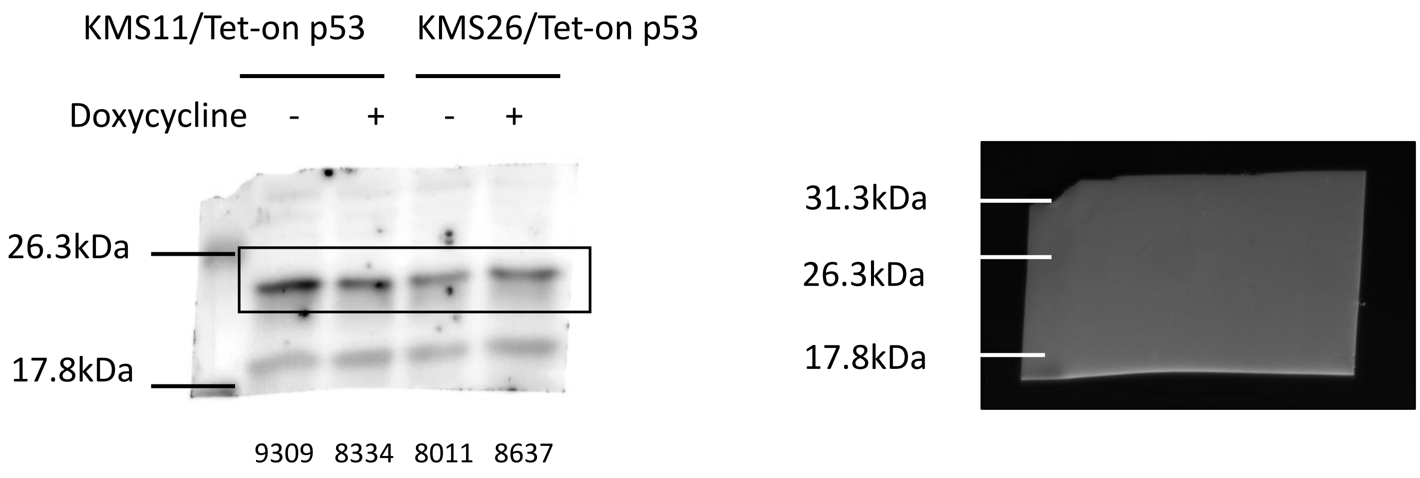


## p53

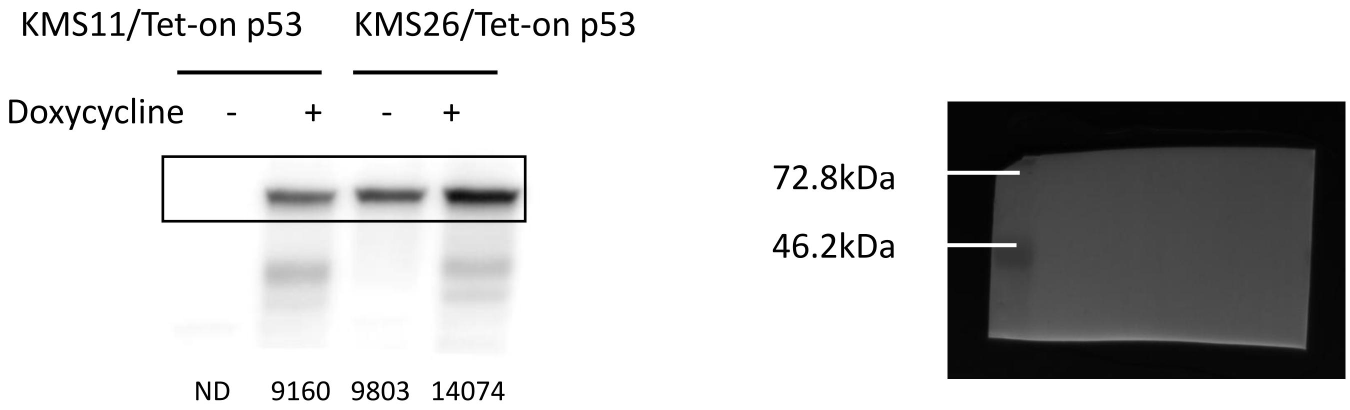


ND; Not detected

# Fig.S2 A,B 48h

## ACTB

KMS11/Tet-on p53 KMS26/Tet-on p53

Doxycycline - + - +

46.2kDa

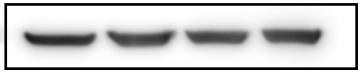

14391 13597 12495 11856

72.8kDa

46.2kDa

31.3kDa

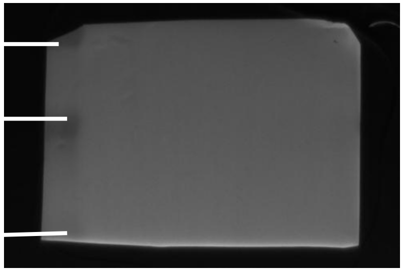

# Fig.S2  A,B 72h

Rac1

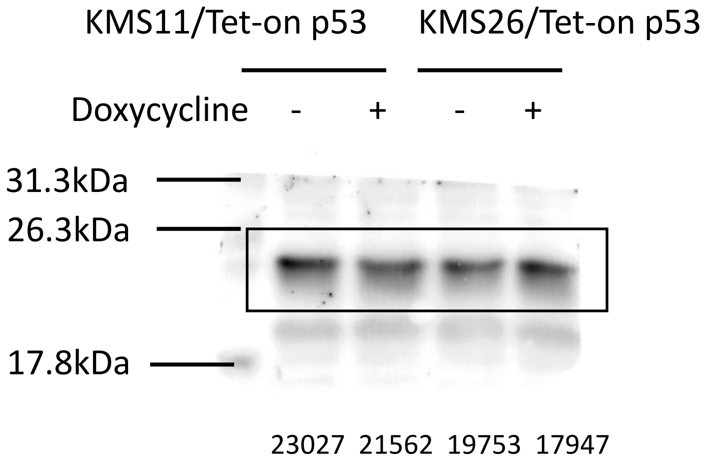


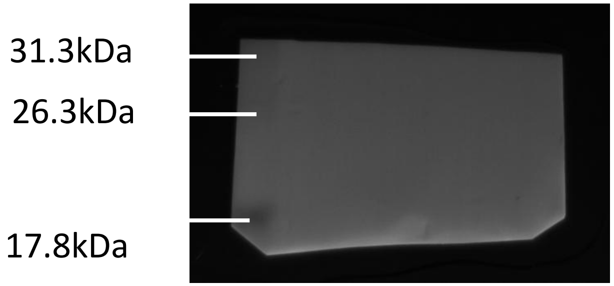

# Fig.S2 A,B 72h

p53

KMS11/Tet-on p53 KMS26/Tet-on p53

Doxycycline - + - +

46.2kDa

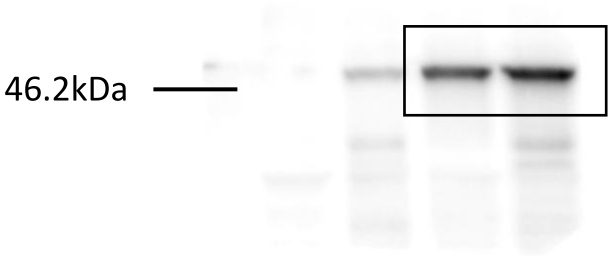

* * 11960 15578

72.8kDa

46.2kDa

31.3kDa

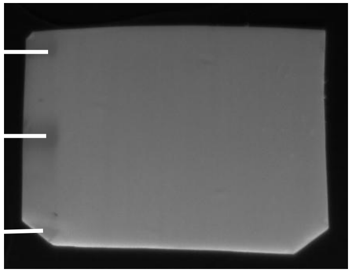

KMS11/Tet-on p53 KMS26/Tet-on p53

Doxycycline - + - +

46.2kDa

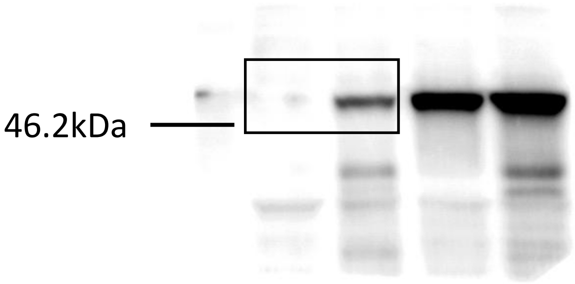

ND 13156 * *

Detected on the same membrane with different exposure times.
Exposure time A: 60sec, B: 200sec.
ND; Not detected, *; No used in results.

# Fig.S2  A,B 72h

ACTB

KMS11/Tet-on p53    KMS26/Tet-on p53

Doxycycline  -  +  -  +

46.2kDa

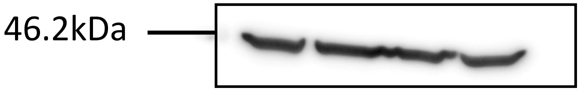

15413  15700  14877  12017

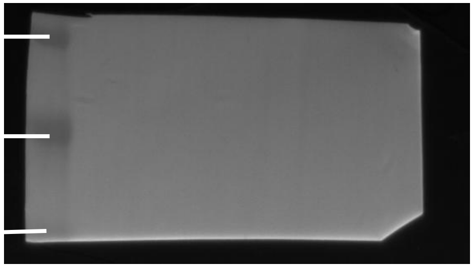

# Fig.S2 C MM.1S 48h, 72h

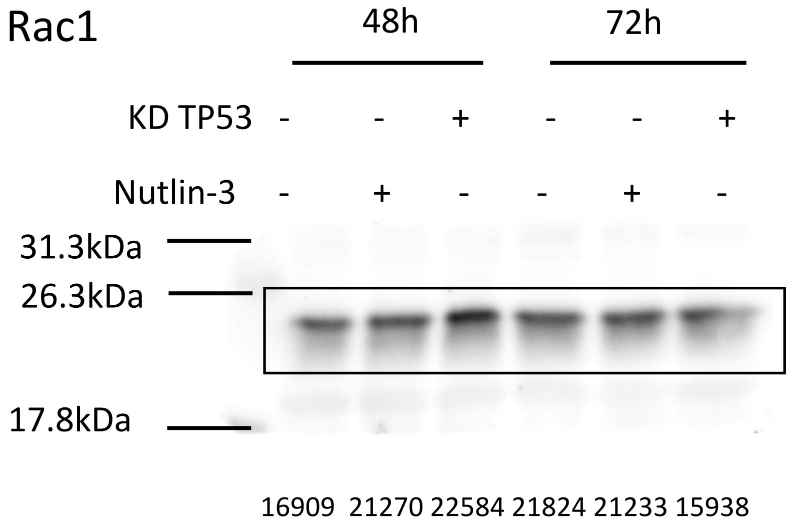


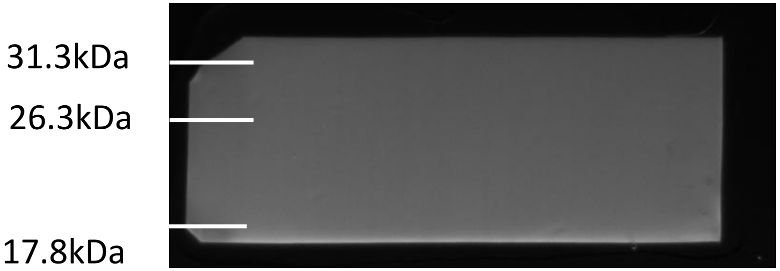


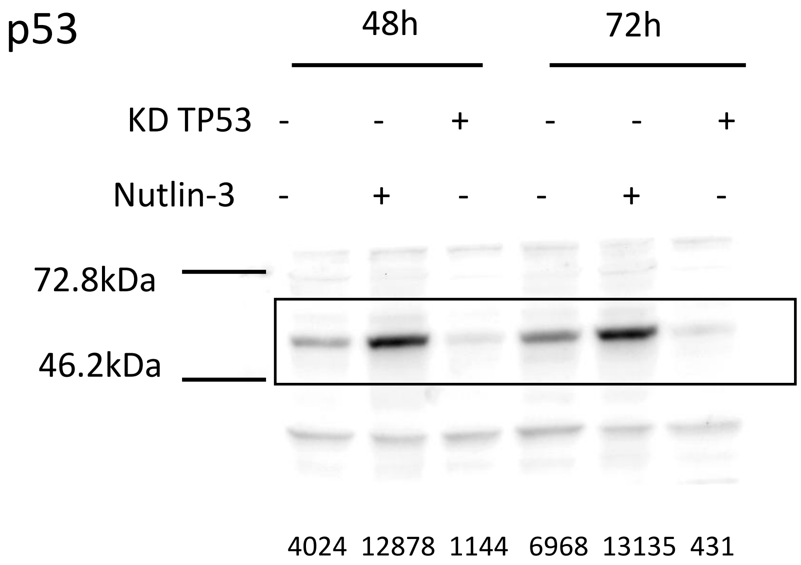


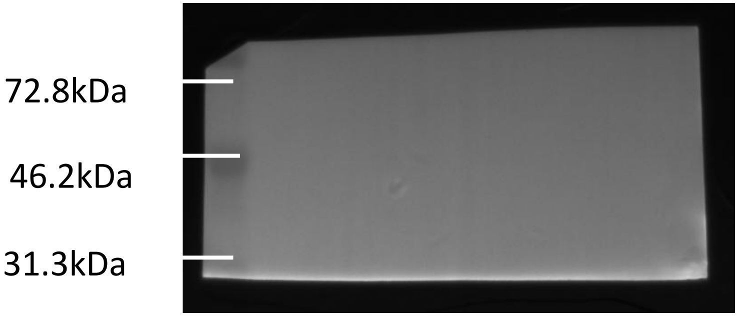

# Fig.S2 C MM.1S 48h, 72h

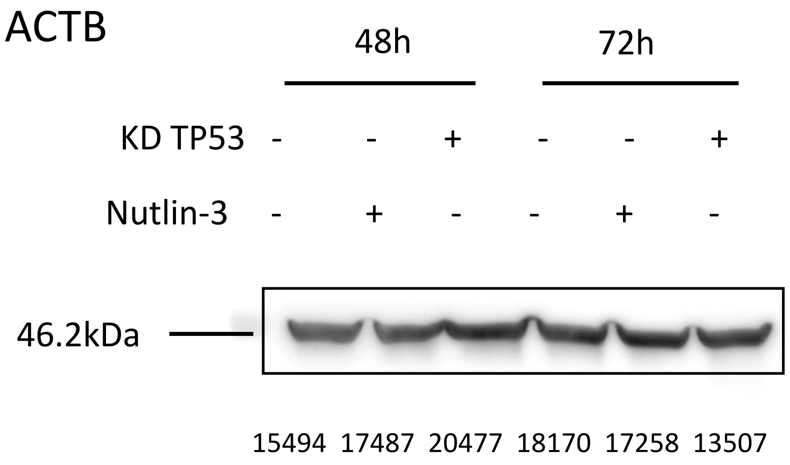


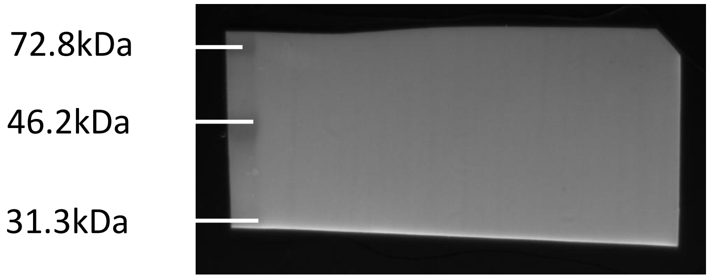

# Fig.S4

## A KMS11 / Tet-on p53 24h

p53

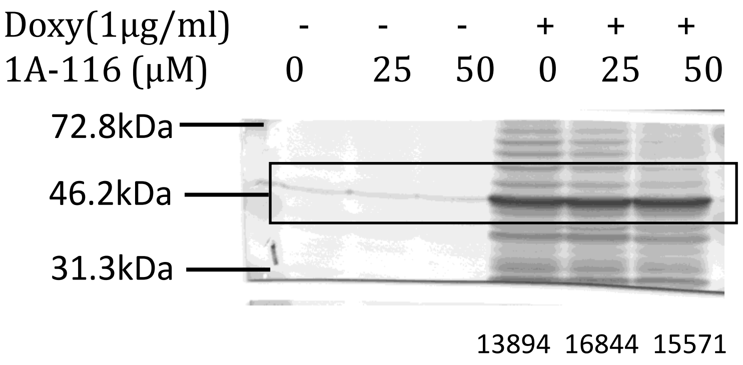


p21

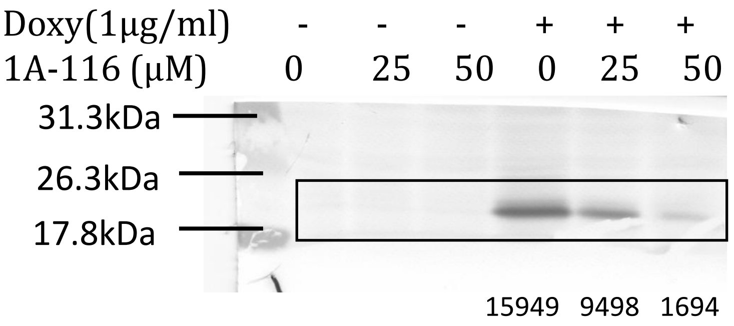


ACTB

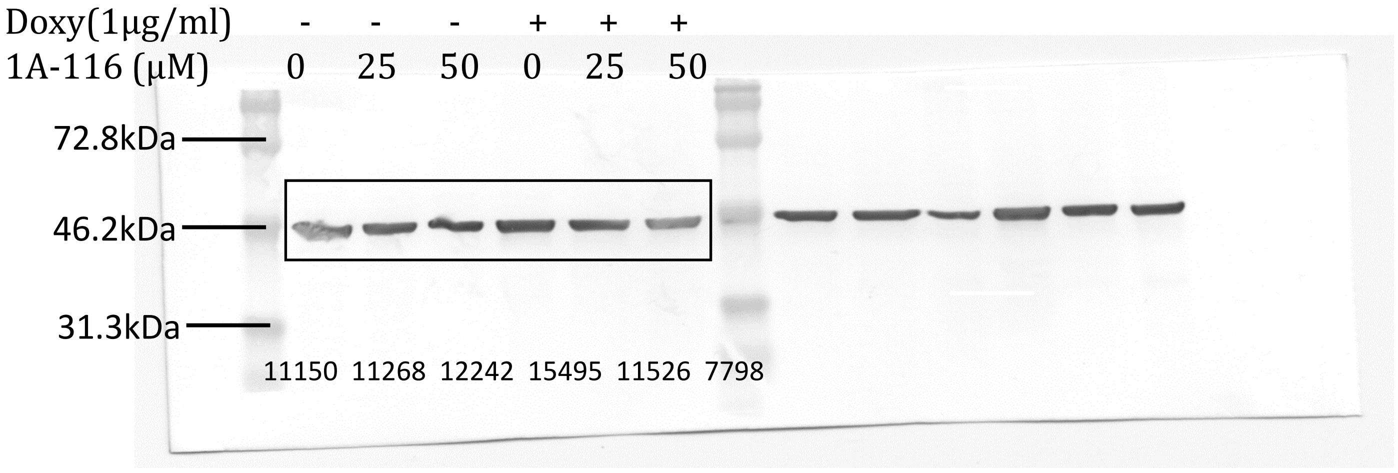

# Fig.S4

## A KMS11 / Tet-on p53 48h

### p53

Doxy(1μg/ml) - - - + + +

1A-116 (μM) 0 25 50 0 25 50

72.8kDa

46.2kDa

31.3kDa

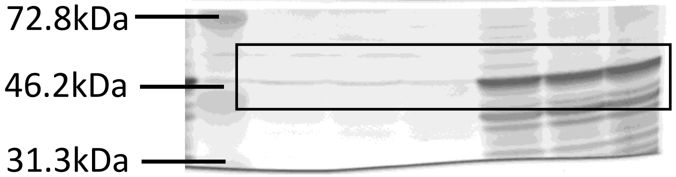

14037 15366 12855

### p21

Doxy(1μg/ml) - - - + + +

1A-116 (μM) 0 25 50 0 25 50

31.3kDa

26.3kDa

17.8kDa

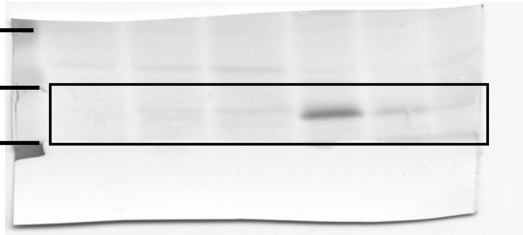

13206 4766 873

### ACTB

Doxy(1μg/ml) - - - + + +

1A-116 (μM) 0 25 50 0 25 50

72.8kDa

46.2kDa

31.3kDa

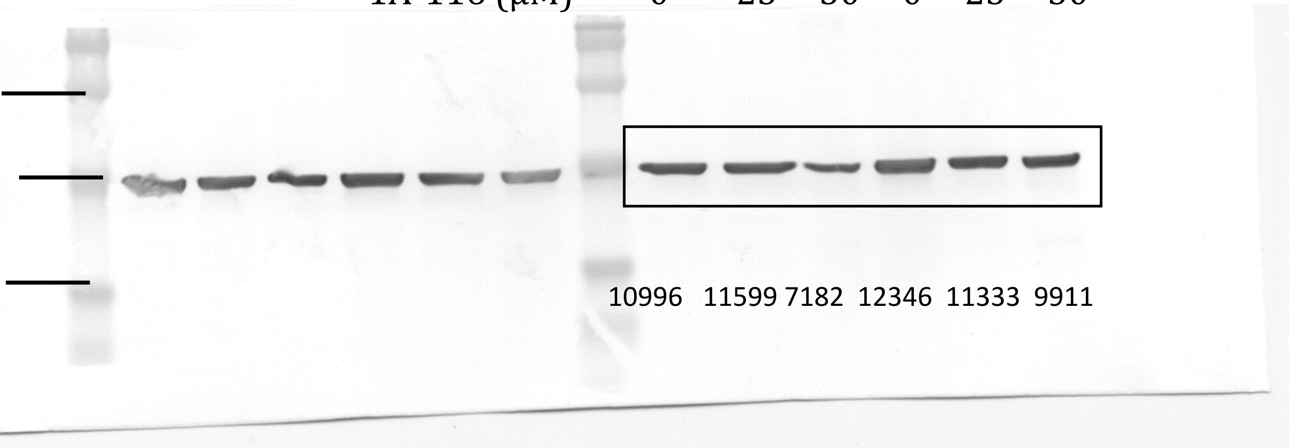

10996 11599 7182 12346 11333 9911

# Fig.S4

## A KMS11 / Tet-on p53 72h

### p53

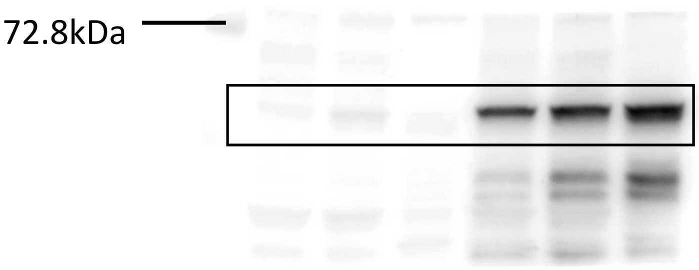


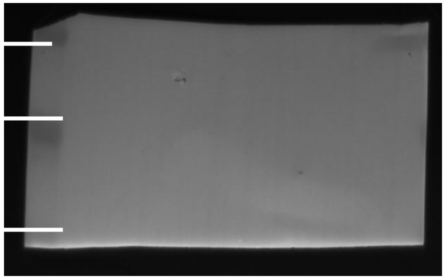


### p21

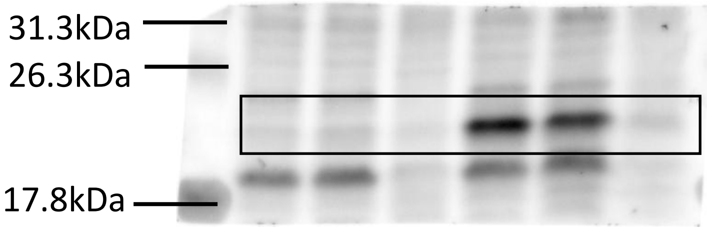


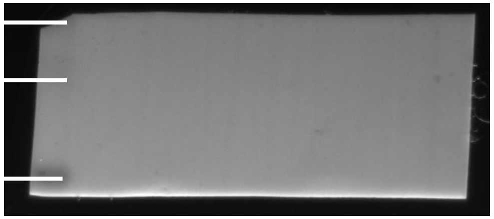

Fig.S4

# A KMS11 / Tet-on p53 72h

ACTB

| | | | | | | |
|---|---|---|---|---|---|---|
| Doxy(1μg/ml) | - | - | - | + | + | + |
| 1A-116 (μM) | 0 | 25 | 50 | 0 | 25 | 50 |

46.2kDa

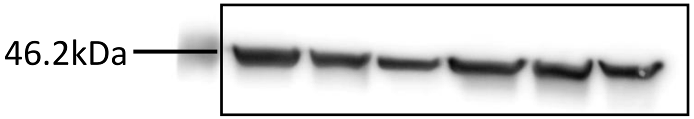

21324 17591 14623 20052 18960 12486

46.2kDa

31.3kDa

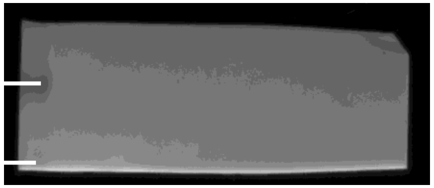

# Fig.S4

## B KMS26 / Tet-on p53 24h

p53

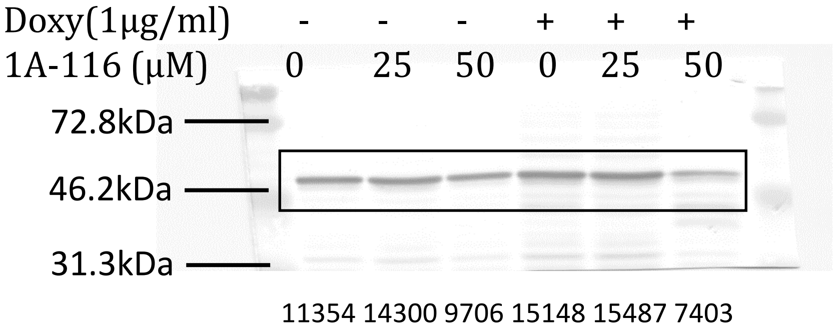


p21

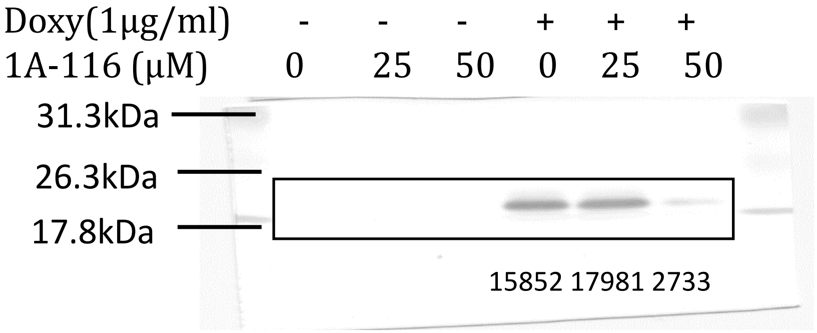


ACTB

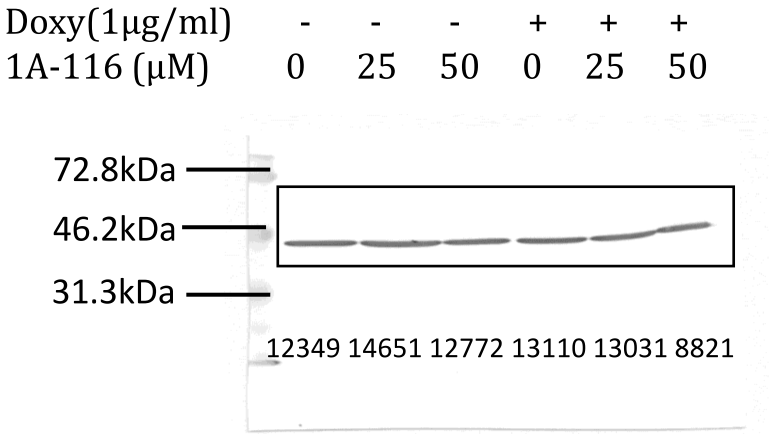

Fig.S4

# A KMS26 / Tet-on p53 48h

## p53

| | | | | | | |
|---|---|---|---|---|---|---|
| Doxy(1μg/ml) | - | - | - | + | + | + |
| 1A-116 (μM) | 0 | 25 | 50 | 0 | 25 | 50 |

72.8kDa

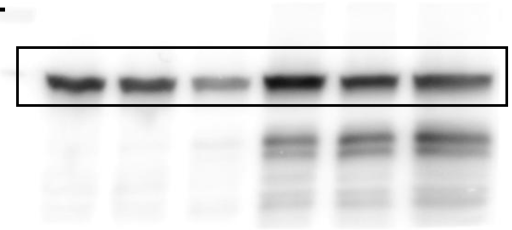

18082 14900 9006 22542 17501 15724

72.8kDa
46.2kDa
31.3kDa

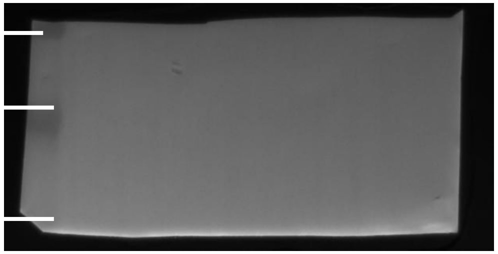

## p21

| | | | | | | |
|---|---|---|---|---|---|---|
| Doxy(1μg/ml) | - | - | - | + | + | + |
| 1A-116 (μM) | 0 | 25 | 50 | 0 | 25 | 50 |

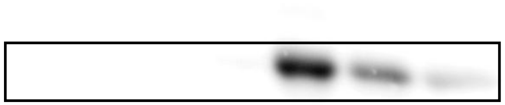

29266 12358 2481

26.3kDa
17.8kDa

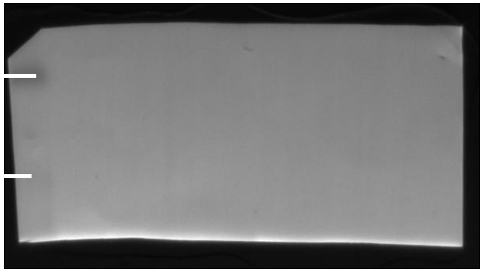

Fig.S4

A KMS26 / Tet-on p53 48h

ACTB

| | | | | | | |
|---|---|---|---|---|---|---|
| Doxy(1μg/ml) | - | - | - | + | + | + |
| 1A-116 (μM) | 0 | 25 | 50 | 0 | 25 | 50 |

46.2kDa

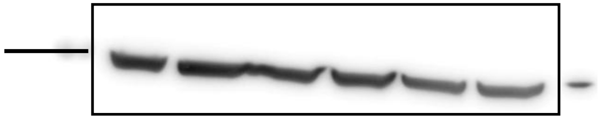

14448 18274 14659 14645 11119 8070

46.2kDa

31.3kDa

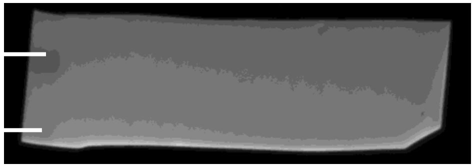

Fig.S4

A KMS26 / Tet-on p53 72h

p53

| | | | | | | |
|---|---|---|---|---|---|---|
| Doxy(1μg/ml) | - | - | - | + | + | + |
| 1A-116 (μM) | 0 | 25 | 50 | 0 | 25 | 50 |

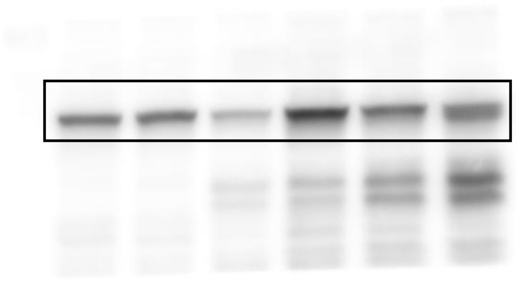

72.8kDa
46.2kDa
31.3kDa

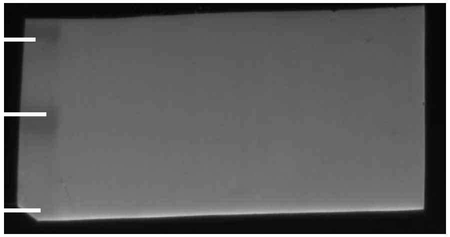

10965 11621 5813 19959 14467 11375

p21

| | | | | | | |
|---|---|---|---|---|---|---|
| Doxy(1μg/ml) | - | - | - | + | + | + |
| 1A-116 (μM) | 0 | 25 | 50 | 0 | 25 | 50 |

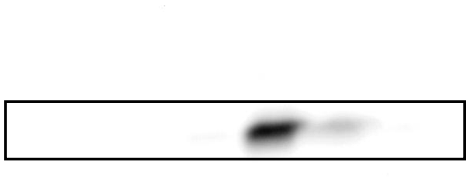

31.3kDa
26.3kDa
17.8kDa

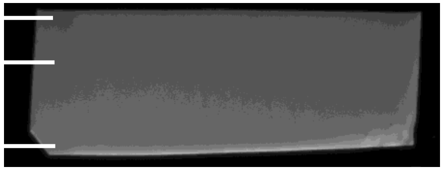

25535 5009

Fig.S4

A KMS26 / Tet-on p53 72h

ACTB

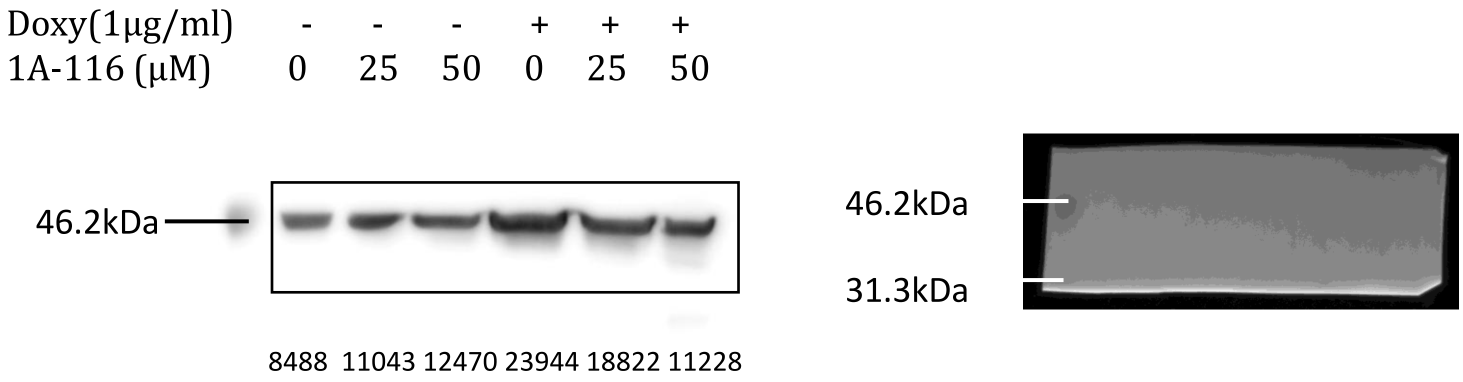

Fig.S4

C MM.1S 24h

p53

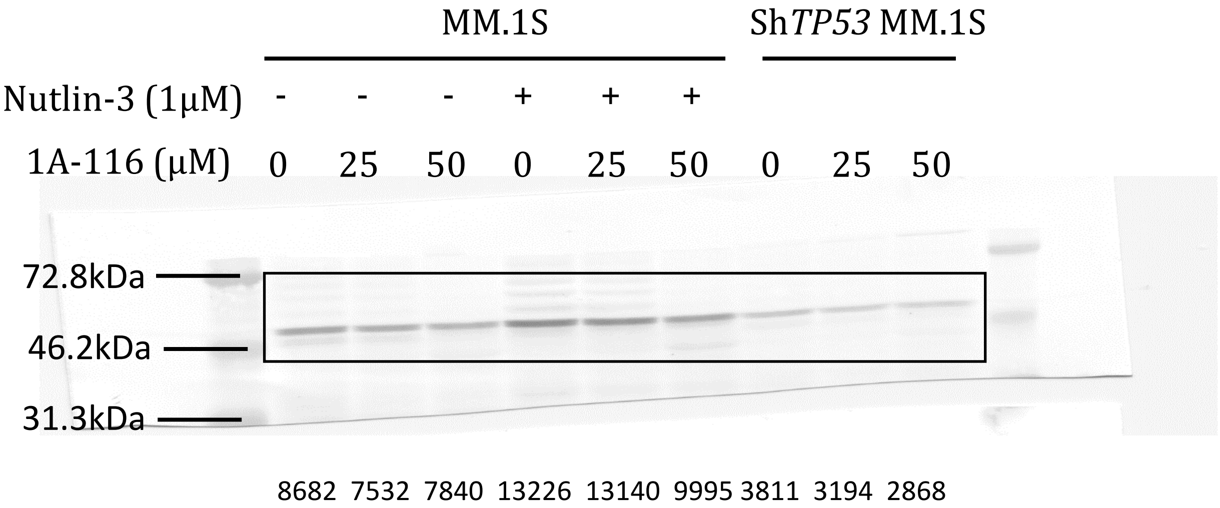


p21

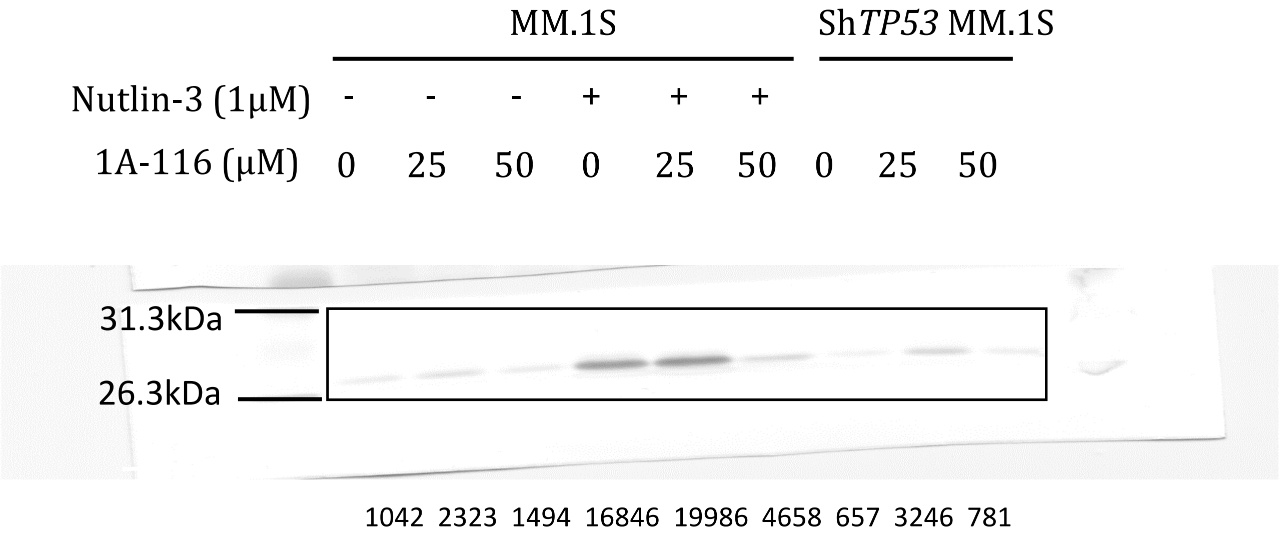

Fig.S4

# C MM.1S 24h

ACTB

| | MM.1S | | | | | | Sh*TP53* MM.1S | | |
|---|---|---|---|---|---|---|---|---|---|
| Nutlin-3 (1μM) | - | - | - | + | + | + | | | |
| 1A-116 (μM) | 0 | 25 | 50 | 0 | 25 | 50 | 0 | 25 | 50 |

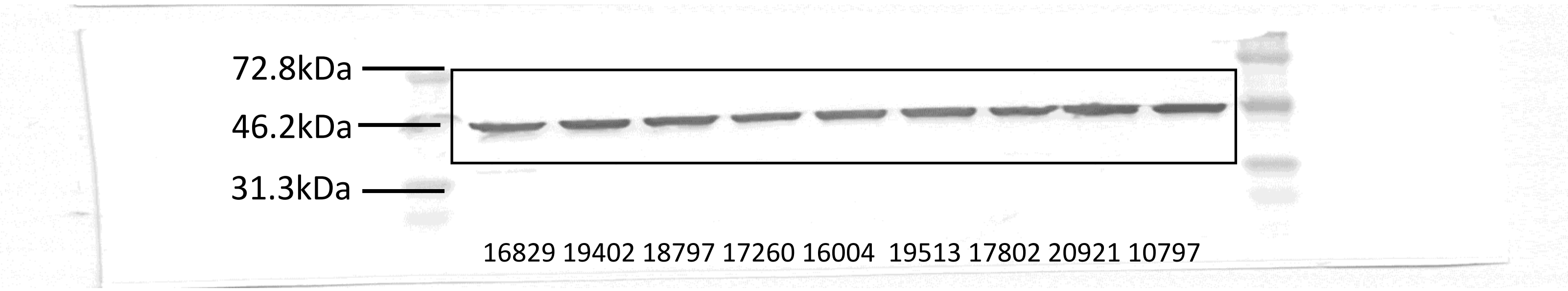

Fig.S4

# C MM.1S 48h

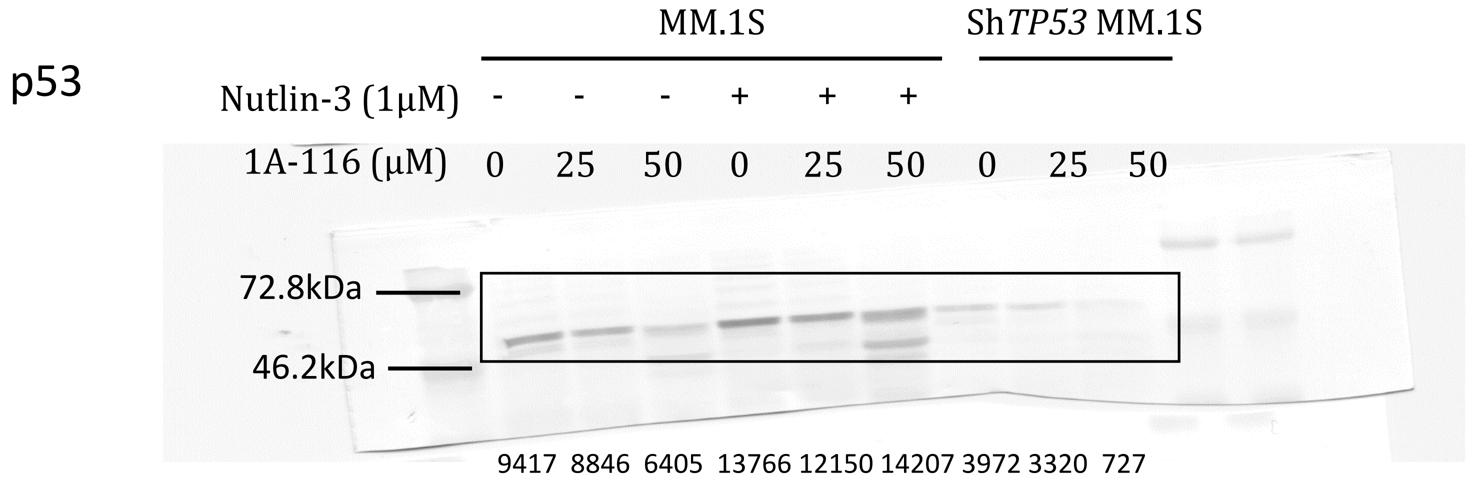


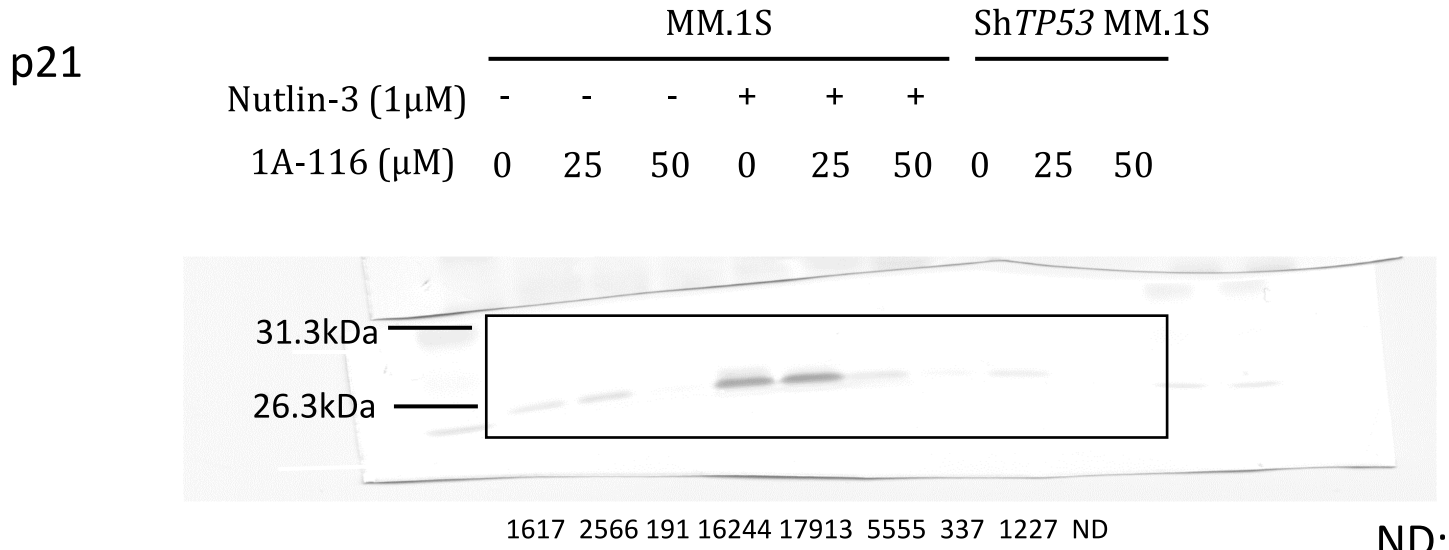


ND; Not detected.

Fig.S4

C MM.1S 48h

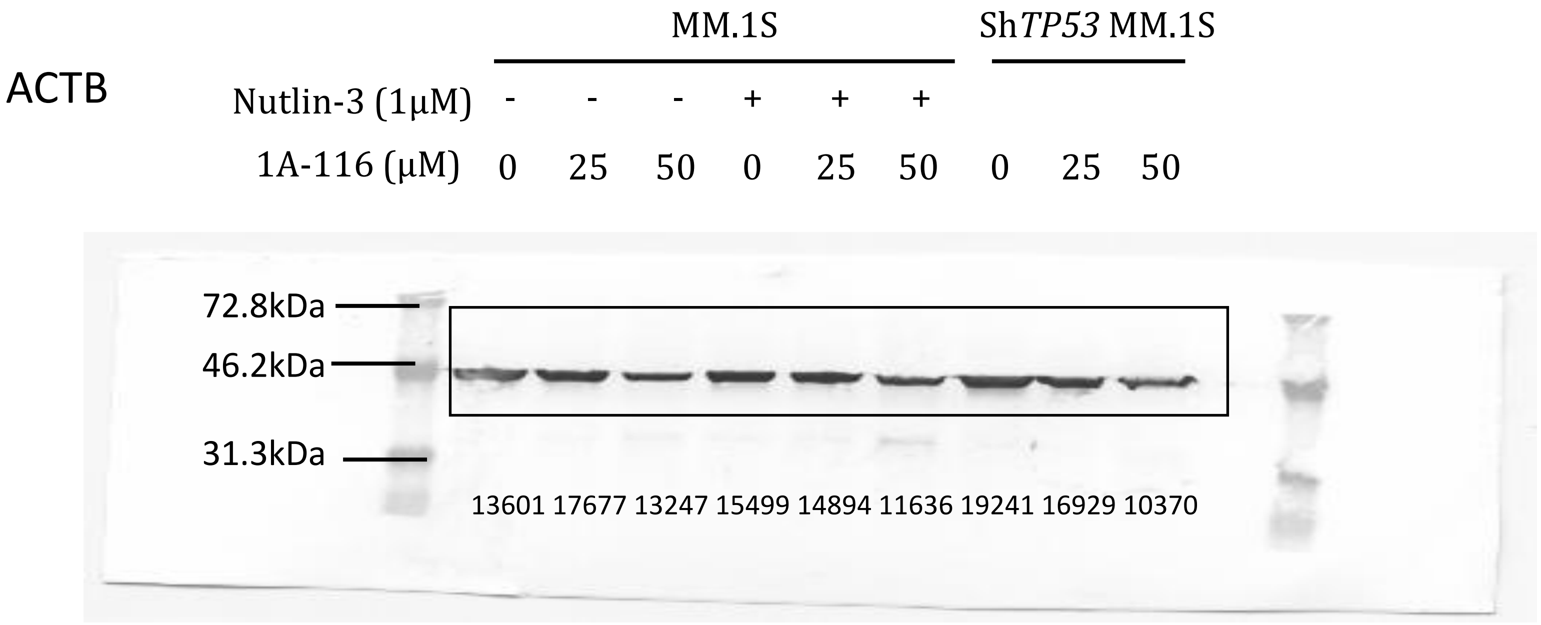

Fig.S4

C MM.1S 72h

p53

MM.1S | Sh*TP53* MM.1S

Nutlin-3 (1μM) - - - + + +

1A-116 (μM) 0 25 50 0 25 50 0 25 50

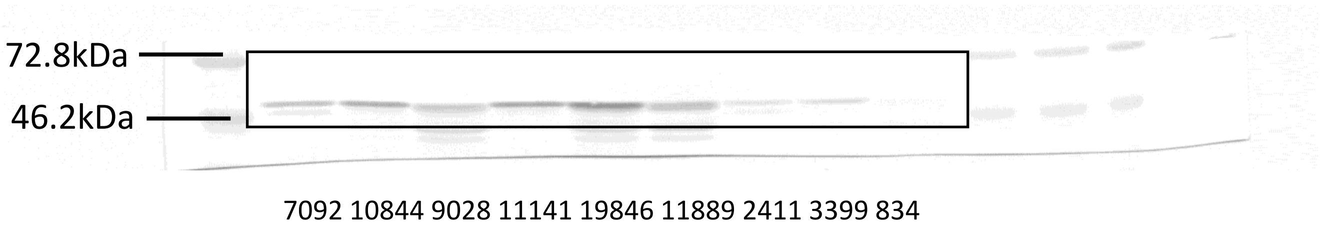


p21

MM.1S | Sh*TP53* MM.1S

Nutlin-3 (1μM) - - - + + +

1A-116 (μM) 0 25 50 0 25 50 0 25 50

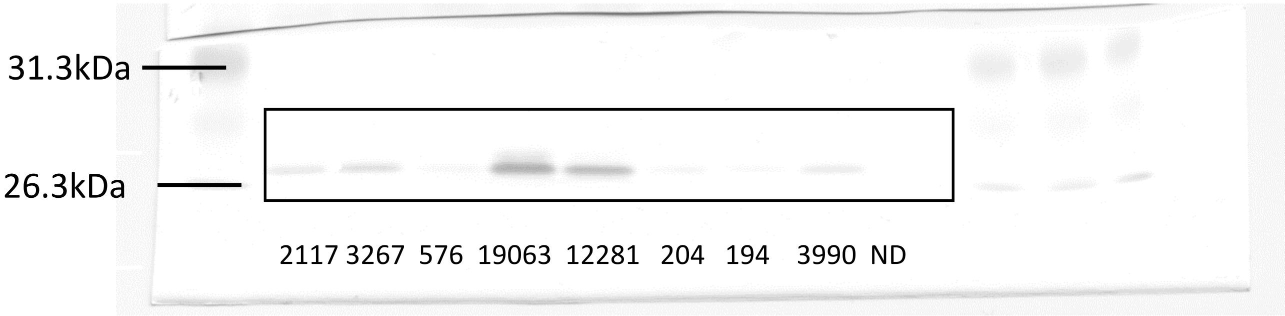

Fig.S4

# C MM.1S 72h

ACTB

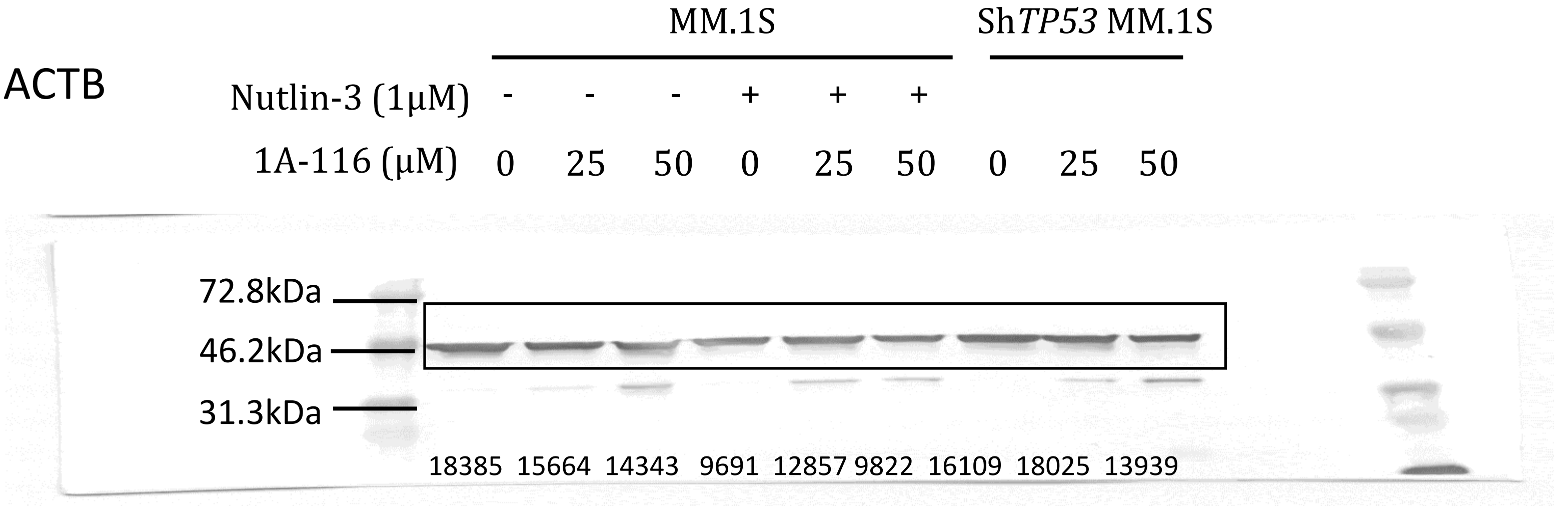

Supplement: Supplementary file 1 [file cancers-17-00461-s001.zip › File S1. Original images of western blotting.pdf]
