# Supplementary material for: Role of Rac1 in p53-Related Proliferation and Drug Sensitivity in Multiple Myeloma
Source: Cancers (Basel). 2025 Jan 29;17(3):461. doi: 10.3390/cancers17030461 (PMC11815915; doi:10.3390/cancers17030461)
Supplement: Supplementary file 1 [file cancers-17-00461-s001.zip › Supplementary Materials.pdf]

Supplementary Materials

Document S1. **Materials and Methods Expression of Wild-type p53 Using Tet-on System**

Doxycycline-inducible lentivirus vector pCW57.1 (plasmid #41393) and R777-E351 Hs.*TP53* (plasmid #70635) encoding wild-type (WT) p53 were purchased from Addgene (Cambridge, MA, USA). p53 complementary DNA (cDNA) was inserted into pCW57.1 using the Gateway® LR Clonase™ Enzyme Mix kit (Thermo Fisher Scientific, Waltham, MA, USA). Pseudo-type viruses were produced by co-transfecting pCW57.1-*TP53*, pCAG-HIVgp, and pCMV-VSV-G-RSV-Rev into 293T cells using Lipofectamine 2000 (Thermo Fisher Scientific)

Document S2. **Materials and Methods RNA Sequencing**

RNA integrity numbers were confirmed to be >7 using the Agilent RNA6000 Pico Kit (5067-1513) in the Agilent 2100 Bioanalyzer (Agilent, Santa Clara, CA, USA). The RNA library was constructed using the NEBNext Ultra Directional RNA Library Prep Kit for Illumina (E7420; NEB, Ipswich, MA, USA), NEBNext Multiplex Oligos for Illumina (E7335 or 7500), and Agencourt AMPure XP (A63881; Beckman Coulter, Brea, CA, USA), following the manufacturer's

protocols. The quality of the amplified cDNA library was determined using the Agilent 2100 Bioanalyzer and high-sensitivity DNA kit (5067-4626; Agilent). RNA sequencing was performed, and raw data were aligned against the human genome reference (hg38) using the DRAGEN RNA pipeline application (Illumina). Differential expression were analyzed using the DRAGEN Differential Expression application (Illumina).

Metascape software first identified all statistically enriched terms (can be GO/KEGG terms, canonical pathways, hall mark gene sets, etc., based on the default choices under Express Analysis or your choice during Custom Analysis), accumulative hypergeometric p-values and enrichment factors were calculated and used for filtering. The remaining significant terms were then hierarchically clustered into a tree based on Kappa-statistical similarities among their gene memberships (similar to what is used in NCI DAVID site). Then 0.3 kappa score was applied as the threshold to cast the tree into term clusters.

Metascape software selected the term with the best p-value within each cluster as its representative term and displayed them in a dendrogram. The heatmap cells are colored by their p-values, white cells indicate the lack of enrichment for that term in the corresponding gene list.

A B

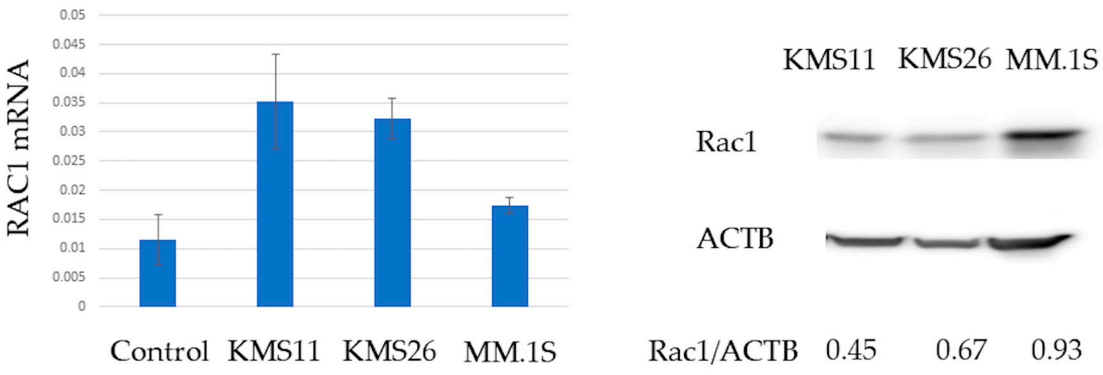

Figure S1 **RAS-related C3 botulinus toxin substrate 1 (*RAC1*) mRNA and protein expression in human myeloma cell lines (HMCLs).** (A) *RAC1* mRNA and (B) Rac1 protein expression in KMS11, KMs26, and MM.1S. Control, *RAC1* mRNA expression of patients in the control group. ACTB, actin beta.

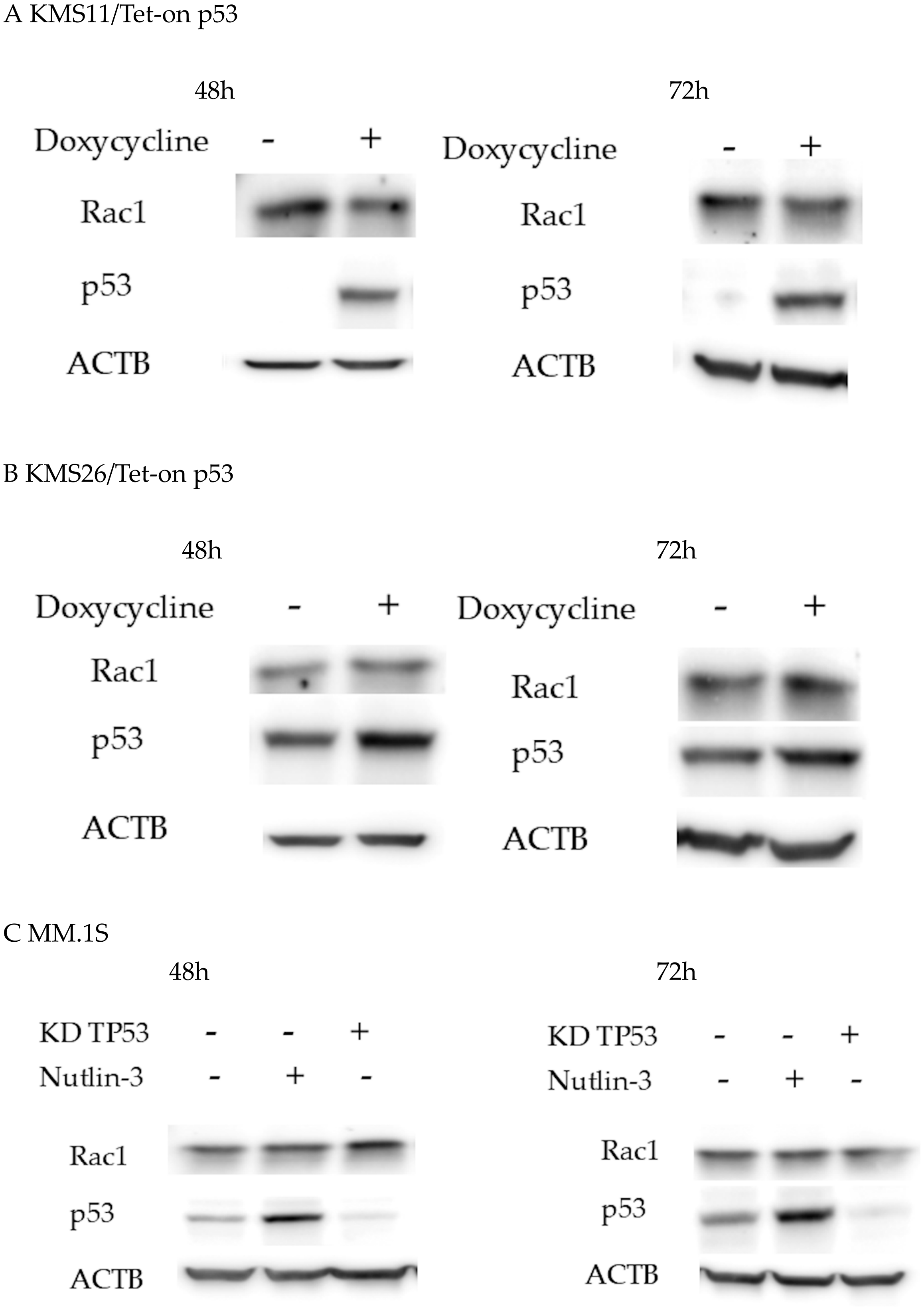

A KMS11/Tet-on p53
48h
72h
Doxycycline - +
Doxycycline - +
Rac1
p53
ACTB
B KMS26/Tet-on p53
48h
72h
Doxycycline - +
Doxycycline - +
Rac1
p53
ACTB
C MM.1S
48h
72h
KD TP53 - - +
Nutlin-3 - + -
KD TP53 - - +
Nutlin-3 - + -
Rac1
p53
ACTB

Figure S2 **RAS-related C3 botulinus toxin substrate 1 (Rac1) protein expression of human myeloma cell lines (HMCLs) across p53 statuses at 48 h and 72 h after treatment.** (A) KMS11/Tet-on p53, (B) KMS26/Tet-on p53, and (C) MM.1S. ND, knockdown. ACTB, actin beta.

A KMS11

B KMS26

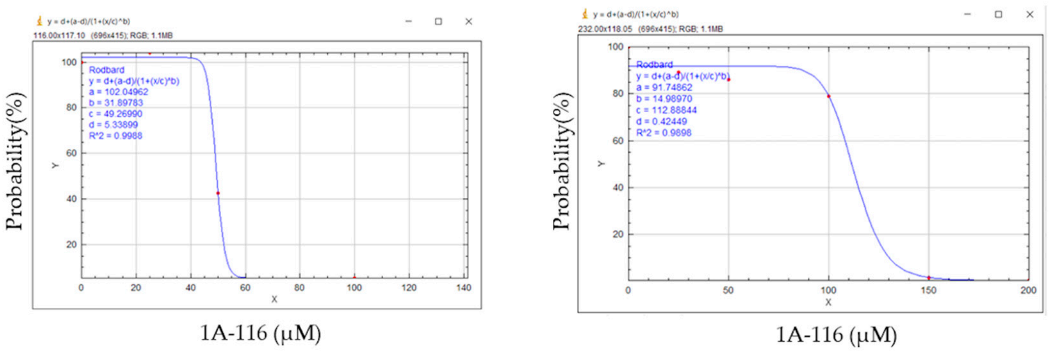

IC50
49.2699*((102.04962-5.33899)/(50-5.33899)-1)^(1/31.89783) = 49.5

IC50
112.88844*((91.74862-0.42449)/(50-0.42449)-1)^(1/14.98970) = 111.6

Figure S3 **The 50 % inhibitory concentration (IC50) of 1A-116 at 72 h, determined using a water-soluble tetrazolium-8 assay and ImageJ software.** (A) KMS11 and (B) KMS26.

A KMS11/Tet-on p53

24h

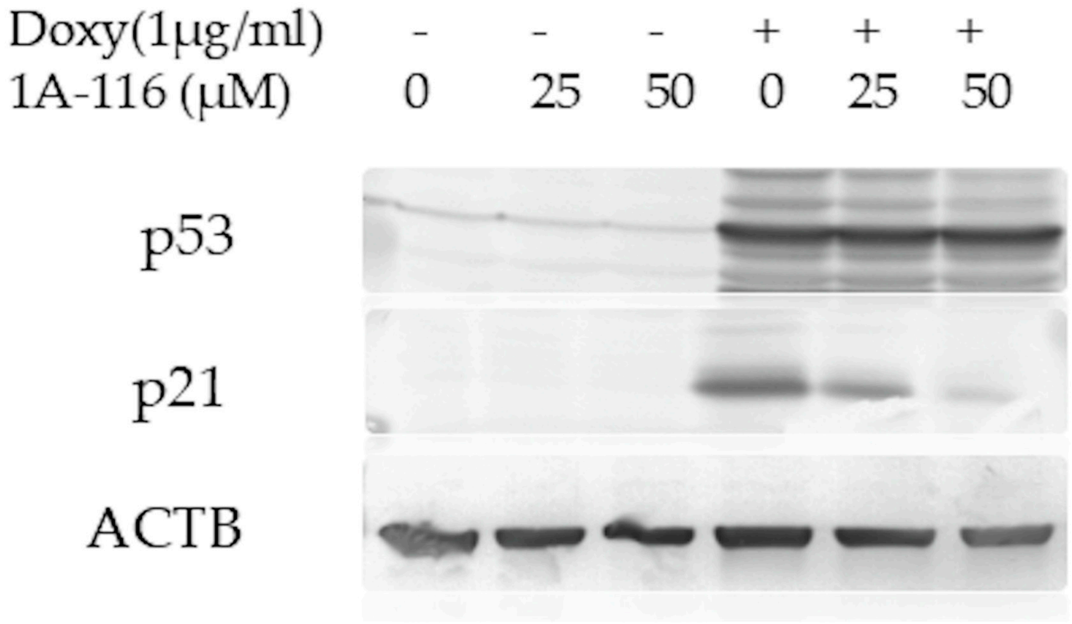

48h

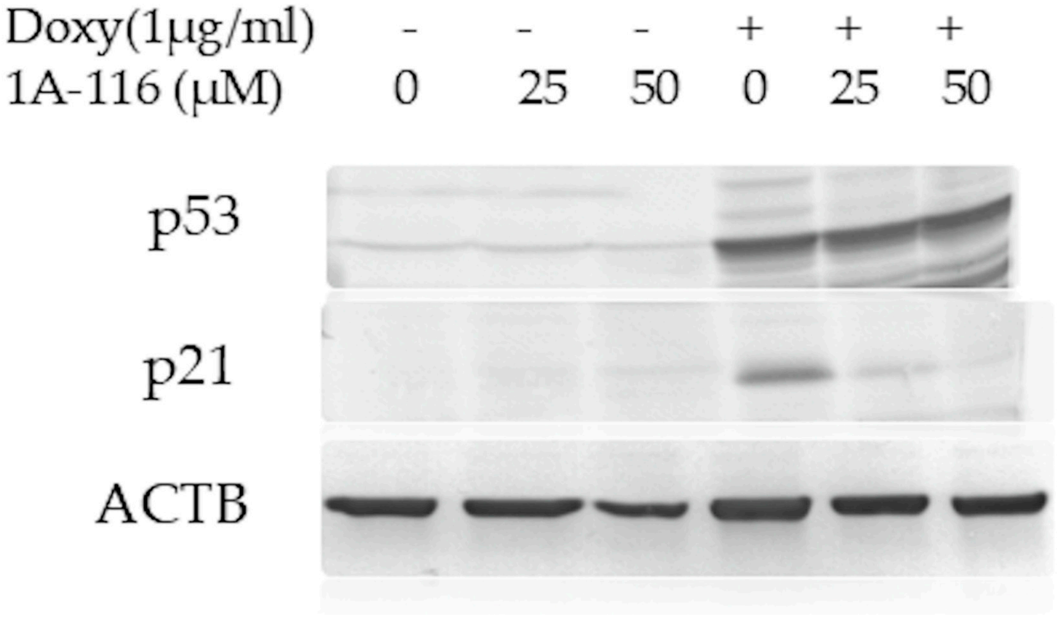

72h

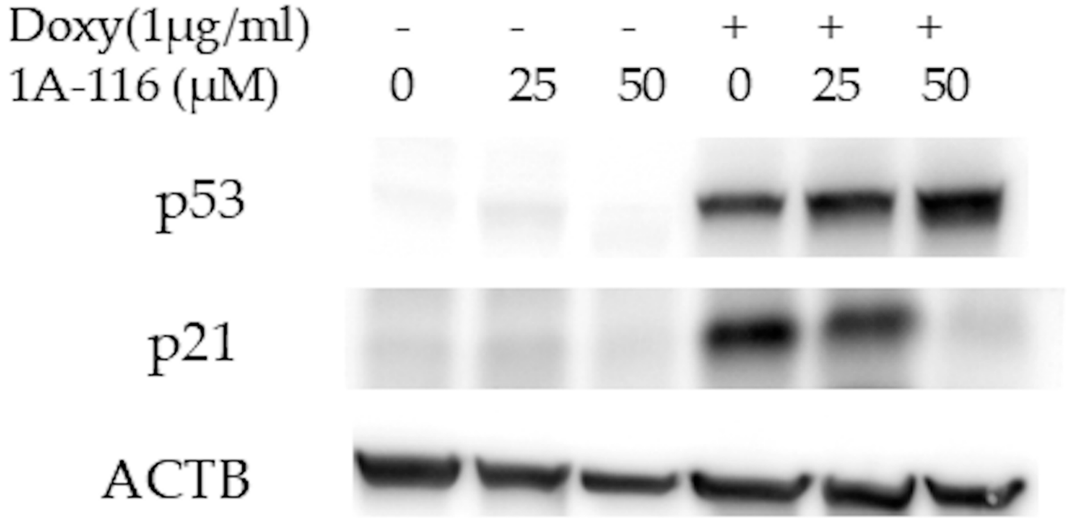

B KMS26/Tet-on p53
24h

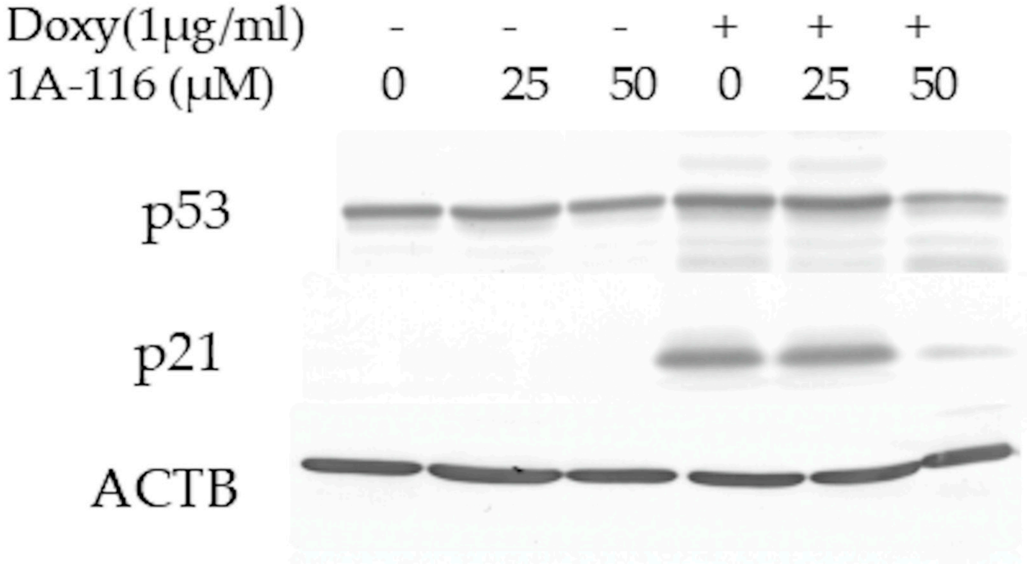

48h

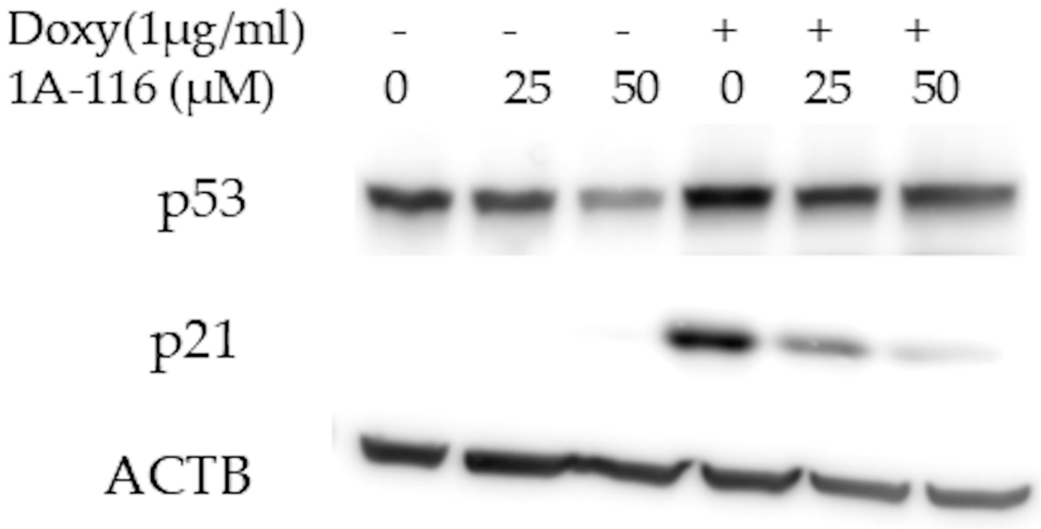

Doxy(1µg/ml) - - - + + +
1A-116 (µM) 0 25 50 0 25 50
p53
p21
ACTB

72h

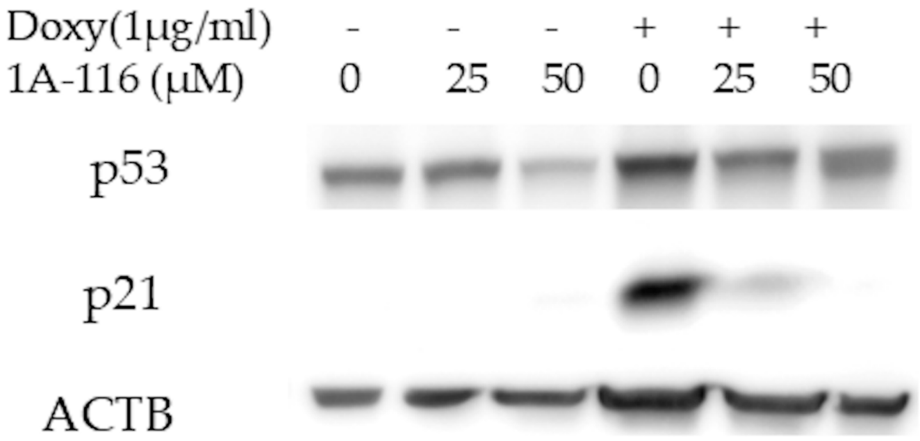

Doxy(1µg/ml) - - - + + +
1A-116 (µM) 0 25 50 0 25 50
p53
p21
ACTB

C

24h

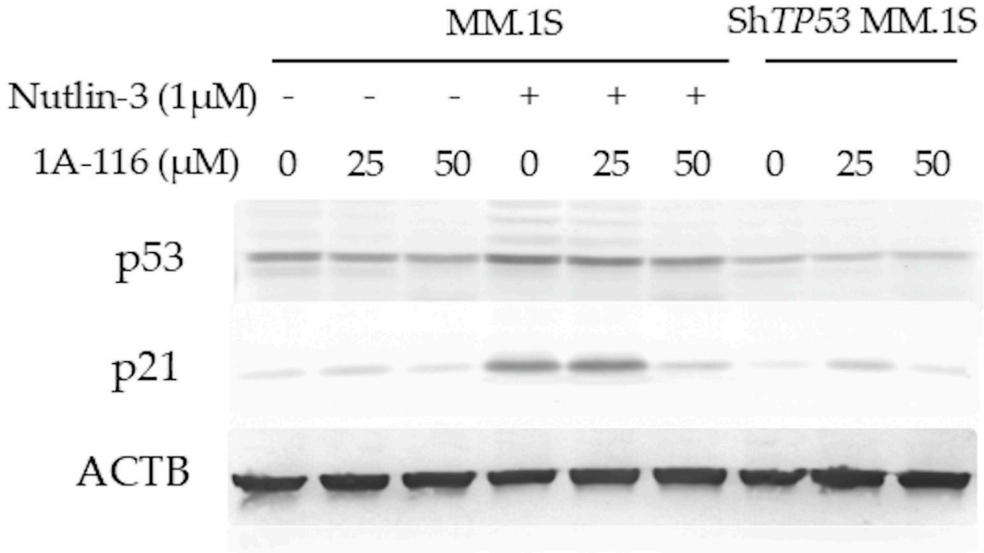
MM.1S
ShTP53 MM.1S
Nutlin-3 (1μM) - - - + + +
1A-116 (μM) 0 25 50 0 25 50 0 25 50
p53
p21
ACTB

48h

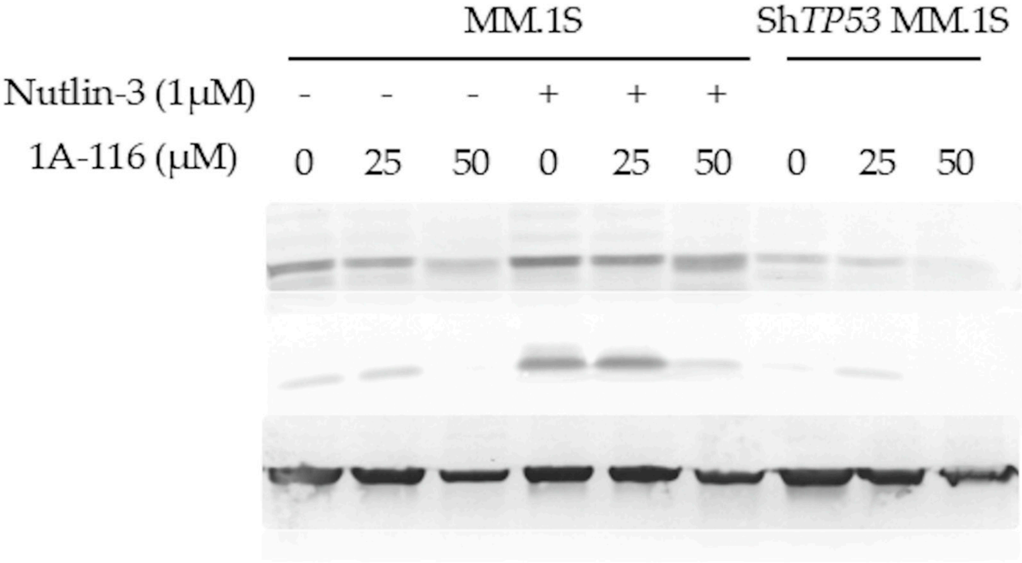
MM.1S
ShTP53 MM.1S
Nutlin-3 (1μM) - - - + + +
1A-116 (μM) 0 25 50 0 25 50 0 25 50

72h

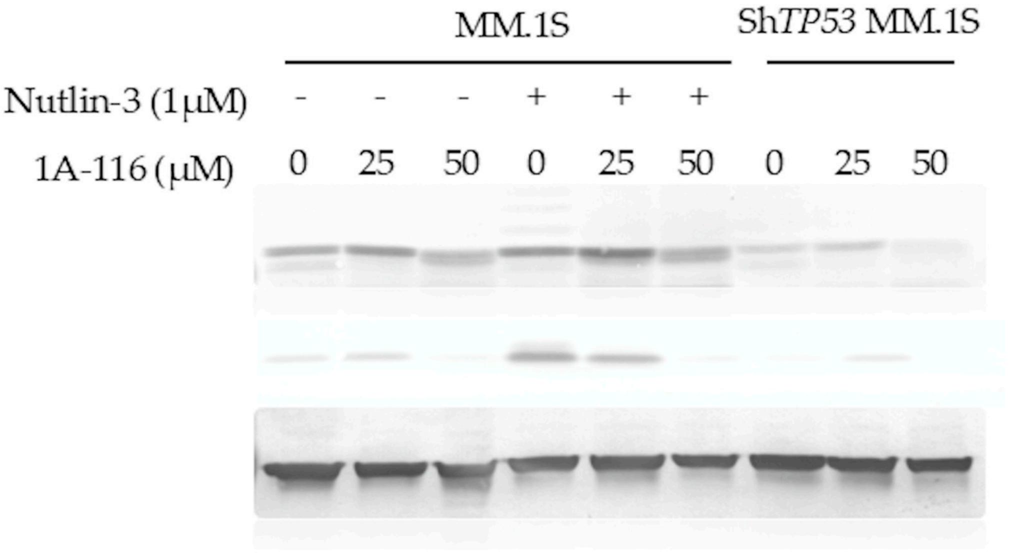

Figure S4 **p53 and p21 protein expression of human myeloma cell lines (HMCLs) at 24h, 48 h, and 72 h after 1A-116 treatment.** (A) KMS11/Tet-on p53, (B) KMS26/Tet-on p53, and (C) MM.1S. Shp53, Short hairpin RNA targeting *TP53*; Doxy, doxycycline; ACTB, actin beta.

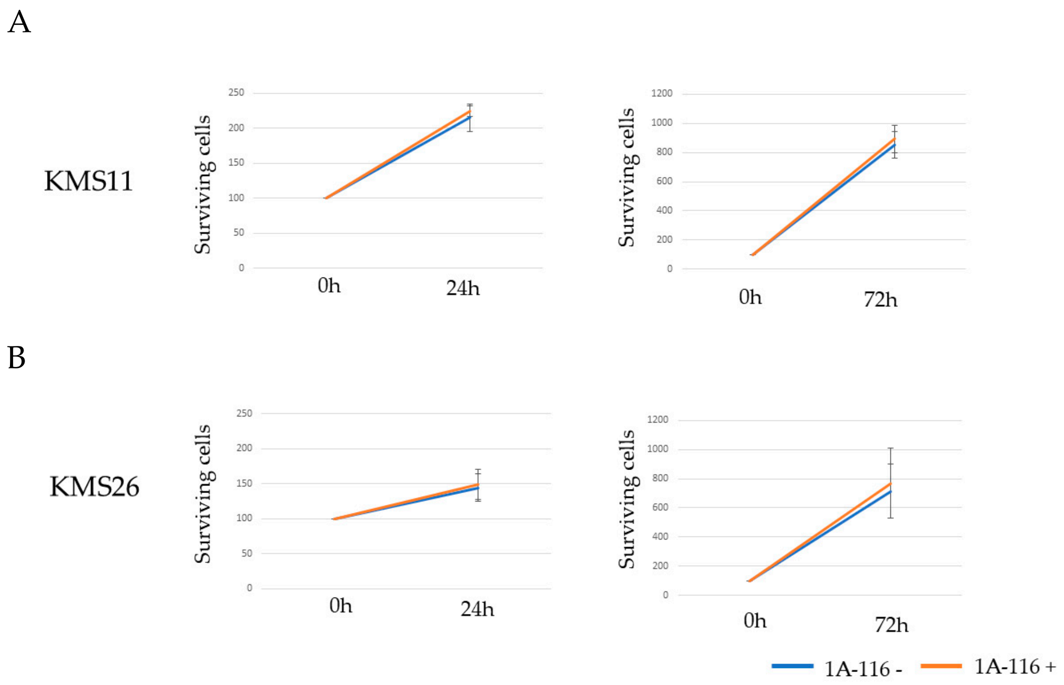

Figure S5 **The proliferation after treatment with 1A-116 25μM determined using a water-soluble tetrazolium-8 assay at 24 h and 72h.** (A) KMS11 and (B) KMS26.

Table S1. Primers used in the study.

Gene		Sequence (5'–3')
TP53	Forward	TCAGCATCTTATCCGAGTGGAA
	Reverse	TGTAGTGGATGGTGGTACAGTCA
CDKN1A	Forward	GAAGACCATGTGGACCTGTCACT
	Reverse	GAAGATCAGCCGGCGTTTG
MDM2	Forward	ATCTACAGGGACGCCATCGAA
	Reverse	CTGAATCCTGATCCAACCAATCAC
RAC1	Forward	CCTGTAGTCGCTTTGCCTATTGA
	Reverse	AGGGTCCCACGCTGTATTCTC
ACTB	Forward	TGGCACCCAGCACAATGAA
	Reverse	CTAAGTCATAGTCCGCCTAGAAGCA

TP53, tumor protein p53; *CDKN1A*, cyclin-Dependent Kinase Inhibitor 1A; *MDM2*, mouse double minute 2 homolog; *RAC1*, Ras-related C3 botulinum toxin substrate 1; *ACTB*, actin beta

Table S2. Antibodies used in the study.

protein	Antibody
ACTB	β-actin (D6A8) Rabbi mAb #8457<br>Cell Signaling Technology, Danvers, MA, USA
p53	p53 (DO-1) Mouse mAb #18032<br>Cell Signaling Technology, Danvers, MA, USA
p21	p21 Waf1/Cip1(12D1) Rabbit mAb #2947<br>Cell Signaling Technology, Danvers, MA, USA
Mdm2	MDM2 (D1V2Z) Rabbit mAb #86934<br>Cell Signaling Technology, Danvers, MA, USA
Rac1	Rac1 Mouse mAb #66122-1-Ig<br>Proteintech, Rosemont, IL, USA
rabbit IgG	anti-rabbit IgG, HRP-linked antibody #7074<br>Cell Signaling Technology, Danvers, MA, USA
mouse IgG	anti-mouse IgG, HRP-linked antibody #7076<br>Cell Signaling Technology, Danvers, MA, USA

ACTB, actin beta; Mdm2, mouse double minute 2; Rac1, Ras-related C3 botulinum toxin substrate 1; IgG, immunoglobulin G.
